# Surfactant Derived From Stearoyl Chloride and Benzimidazole for Micellar‐Promoted Synthesis of *N*‐Aryl‐1,8‐dioxo‐decahydroacridines in Water at Room Temperature

**DOI:** 10.1002/open.202500600

**Published:** 2026-01-28

**Authors:** Saeedeh Asadian, Mohsen Moradian, Javad Safari

**Affiliations:** ^1^ Department of Organic Chemistry Faculty of Chemistry University of Kashan Kashan Iran

**Keywords:** 1,8‐dioxodecahydroacridines, anionic micelles, green chemistry, nanoreactor

## Abstract

In this article, a new anionic surfactant was prepared through a one‐pot reaction of stearoyl chloride and benzimidazole with aluminum chloride under reflux conditions. Its structure was confirmed by ^1^H and ^13^C Nuclear magnetic resonance (NMR) analysis and Fourier transform infrared spectroscopy. The efficacy of the synthesized surfactant system was evaluated in the synthesis of 1,8‐dioxodecahydroacridines through a three‐component reaction involving aniline, 5,5‐dimethylcyclohexan‐1,3‐dione, and aromatic aldehydes. It was demonstrated that with a 0.2 g amount of synthesized surfactant 1,3‐distearoyl‐1H‐benzo[d]imidazolium chloride (DSBimCl), reaction rates could be significantly increased, and the yield in water as solvent without any co‐catalyst reached 96%. Additionally, the method is ecofriendly, minimizing the use of harmful organic solvents and facilitating efficient catalytic processes that are not achievable with traditional organic solvents.

## Graphical abstract

A new anionic surfactant was synthesized via a one‐pot reaction of stearoyl chloride and benzimidazole with aluminum chloride under reflux. The surfactant was effective in synthesizing 1,8‐dioxodecahydroacridines, achieving a 96% yield in water without the need for cocatalysts. This ecofriendly method reduces the use of harmful solvents and enhances catalytic efficiency compared to traditional solvents.

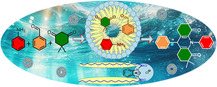

## 1 Introduction

Green chemistry is gaining traction in academia and industry for its emphasis on sustainability in chemical processes. It balances environmental impact and economic benefits by designing products and processes that minimize the use of toxic substances and optimize energy consumption. Over the past twenty years, organic chemists have shifted focus from traditional, efficiency‐driven methods, which often overlooked environmental impacts. The Twelve Principles of Green Chemistry, established by Anastas and Warner in 1998, guide this shift, promoting practices from material design to waste management [1, 2, 3]. Water has emerged as an appealing, ecofriendly solvent in organic reactions, reducing hazardous waste and offering a renewable, nontoxic alternative to petroleum‐based solvents [4, 5, 6, 7, 8, 9, 10, 11].

Since the establishment of the Green Chemistry journal by the Royal Society of Chemistry in 1999, numerous reports have been published on the use of water as a more environmentally friendly solvent in chemical reactions. Aligning with the Twelve Principles of Green Chemistry, the use of water in these reactions pertains to several of these principles [12, 13, 14]. Notably, it can help reduce or eliminate hazardous waste, which primarily arises from organic solvents, by substituting organic solvents with water or facilitating the recycling of water itself [15, 16, 17]. When conducting a reaction in water, organic substrates are typically insoluble and commonly form emulsions. Interestingly, this phenomenon often results in a faster reaction rate compared to organic solvents due to the highly concentrated conditions [18, 19]. The heterogeneous emulsion occurs when hydrophobic organic molecules aggregate into tiny droplets. Reactions then occur within these isolated droplets, thereby accelerating the reaction rate. This process is referred to as an ‘on water’ reaction, which served as the foundational concept for the subsequent development of micellar catalysis in organic synthesis [4, 20, 21].

Micellar catalysis is a promising technology that enables organic reactions to occur more effectively in water. By utilizing the self‐assembly of surfactant molecules into micelles, many reactions can proceed smoothly in an aqueous medium [22, 23, 24]. A surfactant comprises two essential components: a lipophilic (hydrophobic) part and a hydrophilic (water‐attracting) part. This approach addresses the solubility challenges of organic compounds in water by creating a lipophilic core within the micelles that acts as a solvent for the reaction. When surfactant concentrations exceed the critical micelle concentration (CMC), nanoparticles of micelles form in water, acting as nanoreactors where organic reactions can take place [25, 26, 27, 28, 29, 30, 31, 32, 33].

These lipophilic cores are used as hosts for organic combinations. A review of the literature indicates that the synthesis of *N*‐aryl‐1,8‐dioxo‐decahydroacridines is significant for researchers due to its various biological, medicinal, and material science applications [34, 35, 36]. Among the biological properties of acridine diones, anti‐Alzheimer, antifungal, antibacterial, antimicrobial, antimalarial, anticancer, antituberculosis, antidiabetic, and antiviral properties can be mentioned. They are also known to chemists as optical sensitizers in dye lasers in fluorescence chemistry [37, 38, 39, 40, 41, 42, 43]. Various catalysts have been reported for this reaction, such as NH_3_, CeCl_3_·7H_2_O, TEBA, [Hmim]TFA, Fe^3+^‐montmorillonite, Amberlyst‐15 and so on [44, 45, 46, 47].

Nevertheless, these approaches have limitations, including extended reaction times, use of hazardous solvents, elevated temperatures, and low yield of the final product. Additionally, some of these methods have drawbacks, such as the use of expensive catalysts or the requirement for excessive amounts of catalyst. Given the necessity for a streamlined, selective, and high‐yielding synthetic method to promptly supply newly developed compounds for biological testing in potential treatment applications, there is a growing demand for an effective and environmentally friendly method for synthesizing *N*‐aryl‐1,8‐dioxo‐decahydroacridines [48, 49, 50, 51, 52, 53, 54, 55]. The primary motivation behind this innovation is to replace volatile organic solvents with ecofriendly reaction media. This shift aims to address the significant environmental contamination impact of volatile organic solvents, which result from their widespread use and inefficient recovery processes. Water is the most preferred solvent for organic reactions and has become increasingly popular, making it the solvent of choice for many applications [56, 57, 58, 59, 60]. However, the poor solubility of many organic compounds in water can hinder water‐based organic synthesis. To overcome this issue, surfactants have been utilized in aqueous organic reactions [56].

In this contribution, we report a green and practical strategy for synthesizing N‐aryl‐1,8‐dioxo‐decahydroacridines through micellar catalysis in aqueous media, offering a sustainable alternative to conventional synthetic routes. Using surfactant micelles, this method eliminates the need for hazardous organic solvents, operates under mild and energy‐efficient conditions, and yields good to excellent product yields. The protocol marks a significant advance over traditional protocols by minimizing waste generation and relying exclusively on water as the reaction medium.

## 2 Experimental

### 2.1 General

The products were separated and identified based on their physical and spectral characteristics. Nuclear magnetic resonance (^1^H >and ^13^C‐NMR) spectra were recorded using a Bruker Avance‐400 MHz spectrometer with DMSO‐d6 as the NMR solvent. The IR spectra were obtained using an Fourier transform infrared (FT‐IR) Magna 550 apparatus with KBr plates. Melting points were determined using an Electrothermal 9200, and were uncorrected. All purchased chemicals were of high purity and were used without further purification.

### 2.2 Synthesis 1H‐Benzo[d]imidazole (BIM)

First, o‐phenylenediamine (27 g, 0.25 mol) was mixed with formic acid (16 mL, 0.43 mol) in a 250 mL flask. The mixture was heated at 100°C for 2 h. After it was cooled, a 10% sodium hydroxide solution was slowly added dropwise until the mixture became alkaline. The raw product was washed with about 60 mL of cold water under vacuum pump conditions. Finally, a white solid was obtained with a yield of 85% (25.12 g).

### 2.3 Preparation 1,1^′^‐(1H‐3λ4‐Benzo[d]imidazole‐1,3‐diyl)bis(octadecan‐1‐one) (DSBimCl)

To a stirred solution of benzimidazole (1 mmol, 0.12 g) and triethylamine (5 mmol, 0.51 g) in dichloromethane (10 mL), cooled in an ice bath, was added dropwise stearoyl chlorid (2 mmol). The suspension was stirred for 5 h at room temperature, and then washed with water. The organic layer was dried and the solvent evaporated under vacuum. The pure product was obtained as a white powder with a yield of 90% (0.62 g).

### 2.4 Procedure for Preparation of Acridine Derivatives Using DSBimCl as Catalyst

In the reactions of 1,8‐dioxodecahydroacridines catalyzed by DSBimCl, the standard procedure was followed. A solution of 0.02 g (0.03 mmol) DSBimCl in 10 mL of water was prepared, and to this, 1 mmol aniline, 1 mmol aldehyde, and 1  mmol dimedone were added. After stirring the mixture for 30 min at room temperature, while monitoring with thin layer chromatography (TLC) to track the reaction progress, the resulting solid was collected by filtration and washed sequentially with warm water and aqueous ethanol once the reaction was considered complete. Lastly, the solid was dried under reduced pressure to obtain the desired product.

### 2.5 Spectral Data of Products

#### 2.5.1 1,1^′^‐(1H‐3*λ*4‐Benzo[d]imidazole‐1,3‐diyl)bis(octadecan‐1‐one) (DSBimCl)

Yield: 0.62 g, 90%; mp 62–65°C; ^1^H NMR (400 MHz, DMSO‐d6) δ 0.87 (6H, s, 2CH_3_), 1.20–1.24 (56H, m, 14CH_2_), 1.48 (4H, s, 2CH_2_), 2.18 (4H, d, *J* = 6.5 Hz, 2COCH_2_), 7.38–8.21 (4H, d, *J* = 8.0 Hz, ArH), 8.95 (1H, s, NCH),; IR (KBr) 3002, 2848, 1700, 1617, 1469: ^13^C‐NMR (75 MHz, DMSO–d6) δ (ppm): 175.06, 142.13, 129.06, 122.88, 115.63, 46.04, 8.93–34.12.

#### 2.5.2 10‐(3‐Chlorophenyl)−3,3,6,6‐tetramethyl‐9‐phenyl‐2,3,7,8,9,10‐hexahydroacridine‐4,5(1H, 6H)‐dione (4a)

Yield: 0.33 g, 71%; mp 179–181°C; ^1^H NMR (400 MHz, DMSO‐d6) δ 0.73 (6H, s, 2CH_3_), 0.90 (6H, s, 2CH_3_), 1.78 (2H, d, *J* = 7.4 Hz, CH_2_), 2.02 (2H, d, *J* = 7.4 Hz, CH_2_), 2.17–2.24 (4H, m, 2CH_2_), 5.04 (1H, s, CH), 7.10 (1H, t, *J* = 8.1 Hz, ArH), 7.25 (2H, t, *J* = 8.0 Hz, ArH), 7.31–7.33 (2H, m, ArH), 7.43 (1H, s, ArH), 7.63–7.69 (3H, m, ArH); IR (KBr) 3055, 2955, 1642, 1581, 1264, 764 cm^−1^.

#### 2.5.3 10‐(3‐Nitrophenyl)−3,3,6,6‐tetramethyl‐9‐phenyl‐2,3,7,8,9,10‐hexahydroacridine‐4,5(1H, 6H)‐dione (4b)

Yield: 0.45 g, 95%; mp 205–208°C; ^1^H NMR (400 MHz, DMSO‐d6) δ 0.7 (6H, s, 2CH_3_), 0.9 (6H, s, 2CH_3_), IR (KBr) 3048, 2957, 1643, 1589, 1261, 738 cm^−1^. 1.80 (2H, d, *J* = 6.5 Hz, CH_2_), 2.02 (2H, d, *J* = 6.1 Hz, CH_2_), 2.19 (2H, d, *J* = 6.1 Hz, CH_2_), 2.35 (2H, d, *J* = 6.5 Hz, CH_2_), 5.06 (1H, s, CH), 7.10–7.36 (6H, m, ArH), 7.90 (1H, t, *J* = 7.8 Hz,, ArH), 8.42 (2H, d, *J* = 7.7 Hz, ArH);

#### 2.5.4 10‐(4‐Bromophenyl)−3,3,6,6‐tetramethyl‐9‐phenyl‐2,3,7,8,9,10‐hexahydroacridine‐4,5(1H, 6H)‐dione (4c)

Yield: 0.48 g, 95%; mp 302–303°C; ^1^H NMR (400 MHz, DMSO‐d6) δ = 0.72 (6H, s, 2CH_3_), 0.90 (6H, s, 2CH_3_), 1.78 (2H, d, *J* = 6.8 Hz, CH_2_), 2.01 (2H, d, *J* = 7.0 Hz, CH_2_), 2.19 (2H, d, *J* = 7.0 Hz, CH_2_), 2.22 (2H, d, *J* = 6.8 Hz, CH_2_), 5.04 (1H, s, CH), 7.10 (1H, t, *J* = 8.0 Hz, ArH), 7.22–7.26 (3H, m, ArH), 7.31 (2H, d, *J* = 7.8 Hz, ArH), 7.40 (1H, s, ArH), 7.82 (2H, d, *J* = 7.8 Hz, ArH); IR (KBr) 3054, 2952, 1644, 1579, 1264, 743 cm^−1^.

#### 2.5.5 10‐(4‐Chlorophenyl)−3,3,6,6‐tetramethyl‐9‐phenyl‐2,3,7,8,9,10‐hexahydroacridine‐4,5(1H, 6H)‐dione (4d)

Yield: 0.45 g, 98%; mp 219–221°C; ^1^H NMR (400 MHz, DMSO‐d6) δ = 0.72 (6H, s, 2CH_3_), 0.90 (6H, s, 2CH_3_), 1.79 (2H, d, *J* = 7.4 Hz, CH_2_), 2.02 (2H, d, *J* = 7.4 Hz, CH_2_), 2.18 (2H, d, *J* = 7.1 Hz, CH_2_), 2.21 (2H, d, *J* = 7.1 Hz, CH_2_), 5.04 (1H, s, CH), 7.08–7.10 (1H, m, ArH), 7.12–7.30 (5H, m, ArH), 7.32–7.68 (1H, m, ArH), 7.69 (2H, d, *J* = 8.1 Hz, ArH); IR (KBr) 3055, 2952, 1640, 1580, 1263, 764 cm^−1^.

#### 2.5.6 10‐(2‐Chlorophenyl)−3,3,6,6‐tetramethyl‐9‐phenyl‐2,3,7,8,9,10‐hexahydroacridine‐4,5(1H, 6H)‐dione (4e)

Yield: 0.43 g, 95%; mp 187–189°C; ^1^H NMR (400 MHz, DMSO‐d6) δ = 0.74 (6H, s, 2CH_3_), 0.87 (6H, s, 2CH_3_), 1.50 (2H, d, *J* = 6.4 Hz, CH_2_), 1.98 (2H, d, *J* = 6.8 Hz, CH_2_), 2.19 (2H, *J* = 6.4 Hz, CH_2_), 2.23 (2H, *J* = 6.8 Hz, CH_2_), 5.01 (1H, s, CH), 7.07 (1H, t, *J* = 7.8 Hz, ArH), 7.21 (2H, t, *J* = 8.1 Hz, ArH), 7.45 (2H, d, *J* = 8.0 Hz, ArH), 7.57–7.66 (3H, m, ArH), 7.79–7.82 (1H, m, ArH); IR (KBr) 3028, 2954, 1642, 1579, 1258, 792 cm^−1^.

#### 2.5.7 9‐(4‐(Dimethylamino)phenyl)−3,3,6,6‐tetramethyl‐10‐phenyl‐2,3,7,8,9,10‐hexahydroacridine‐4,5(1H, 6H)‐dione (4f)

Yield: 0.42 g, 90%; mp 160–171°C; ^1^H NMR (400 MHz, DMSO‐d6) δ = 1.04 (12H, s, 4CH_3_), 2.31 (8H, s, 4CH_2_), 2.81 (6H, s, 2CH_3_), 5.77 (1H, s, CH), 6.53–6.61 (3H, m, ArH), 6.67–6.98 (6H, m, ArH); IR (KBr) 2960, 2928, 1670, 1594, 1254, 812 cm^−1^.

#### 2.5.8 9‐(4‐Nitrophenyl)−3,3,6,6‐tetramethyl‐10‐phenyl‐2,3,7,8,9,10‐hexahydroacridine‐4,5(1H, 6H)‐dione (4g)

Yield: 0.42 g, 90%; mp 280–283°C; ^1^H NMR (400 MHz, DMSO‐d6) δ = 0.70 (6H, s, 2CH_3_), 0.88 (6H, s, 2CH_3_), 1.78 (2H, d, *J* = 6.1 Hz, CH_2_), 2.01 (2H, d, *J* = 6.3 Hz, CH_2_), 2.20 (2H, d, *J* = 6.1 Hz, CH_2_), 2.24 (2H, d, *J* = 6.3 Hz, CH_2_), 5.14 (1H, s, CH), 7.50 (2H, d, *J* = 8.0 Hz, ArH), 7.57–7.64 (5H, m, ArH), 8.15–8.17 (2H, d, *J* = 8.0 Hz, ArH); IR (KBr) 3063, 2954, 1638, 1585, 1222, 788 cm^−1^.

#### 2.5.9 9‐(2,4‐Dichlorophenyl)−3,3,6,6‐tetramethyl‐10‐phenyl‐2,3,7,8,9,10‐hexahydroacridine‐4,5(1H, 6H)‐dione (4h)

Yield: 0.46 g, 93%; mp 288–290°C; ^1^H NMR (400 MHz, DMSO‐d6) δ = 0.74 (6H, s, 2CH_3_), 0.85 (6H, s, 2CH_3_), 1.75 (2H, d, *J* = 14.0 Hz, CH_2_), 1.96 (2H, d, *J* = 14.1 Hz, CH_2_), 2.13 (2H, d, *J* = 9.2 Hz, CH_2_), 2.17 (2H, *J* = 9.1 Hz, CH_2_), 5.24 (1H, s, CH), 7.33–7.35 (1H, m, ArH), 7.40–7.44 (1H, m, ArH), 7.56–7.58 (1H, m, ArH), 7.59–7.64 (1H, m, ArH); IR (KBr) 3057, 2953, 1646, 1580, 1265, 769 cm^−1^.

#### 2.5.10 10‐(4‐Nitrophenyl)−3,3,6,6‐tetramethyl‐9‐phenyl‐2,3,7,8,9,10‐hexahydroacridine‐4,5(1H, 6H)‐dione (4i)

Yield: 0.43 g, 91%; mp 248–250°C; ^1^H NMR (400 MHz, DMSO‐d6) δ = 0.72 (6H, s, 2CH_3_), 0.88 (6H, s, 2CH_3_), 1.78 (2H, d, *J* = 6.5 Hz, CH_2_), 2.02 (2H, d, *J* = 6.5 Hz, CH_2_), 2.18–2.23 (4H, m, 2CH_2_), 5.05 (1H, s, CH), 7.07–7.33 (5H, m, ArH), 7.75 (2H, d, *J* = 7.8 Hz, ArH), 8.45 (2H, d, *J* = 7.8 Hz, ArH); IR (KBr) 3068, 2956, 1641, 1583, 1221, 762 cm^−1^.

#### 2.5.11 10‐(4‐Bromophenyl)−3,3,6,6‐tetramethyl‐9‐(3‐nitrophenyl)−2,3,7,8,9,10‐hexahydroacridine‐4,5(1H, 6H)‐dione (4j)

Yield: 0.53 g, 96%; mp 291–294°C; ^1^H NMR (400 MHz, DMSO‐d_6_) δ = 0.72 (6H, s, 2CH_3_), 0.90 (6H, s, 2CH_3_), 1.78 (2H, d, *J* = 11.2 Hz, CH_2_), 2.01 (2H, d, *J* = 9.0 Hz, CH_2_), 2.18 (2H, *J* = 11.2 Hz, CH_2_), 2.22 (2H, *J* = 6.3 Hz, CH_2_), 5.04 (1H, s, CH), 7.10 (1H, t, *J* = 8.2 Hz, ArH), 7.22–7.26 (2H, m, ArH), 7.31 (2H, d, *J* = 8.2 Hz, ArH), 7.40 (1H, s, ArH), 7.82 (2H, d, *J* = 8.1 Hz, ArH); IR (KBr) 3043, 2958, 1641, 1588, 1269, 742 cm^−1^.

#### 2.5.12 10‐(4‐Bromophenyl)−3,3,6,6‐tetramethyl‐9‐(5‐bromo‐2‐hydroxyphenyl)−2,3,7,8,9,10‐hexahydroacridine‐4,5(1H, 6H)‐dione (4k)

Yield: 0.58 g, 96%; mp 207–210°C; ^1^H NMR (400 MHz, DMSO‐d6) δ = 0.88 (6H, s, 2CH_3_), 0.97 (3H, s, CH_3_), 1.04 (3H, s, CH_3_), 2.03 (2H, d, *J* = 9.8 Hz, CH_2_), 2.15–2.35 (6H, m, 3CH_2_), 5.03 (1H, s, CH), 6.95–7.29 (3H, m, ArH), 7.38 (1H, d, *J* = 8.0 Hz, ArH), 7.57 (1H, d, *J* = 8.3 Hz, ArH), 7.66 (1H, d, *J* = 8.4 Hz, ArH), 7.89 (1H, s, ArH), 8.93 (1H, s, OH); IR (KBr) 3100, 2958, 1614, 1568, 1269, 740 cm^−1^.

#### 2.5.13 9‐(3‐Nitrophenyl)−3,3,6,6‐tetramethyl‐10‐phenyl‐2,3,7,8,9,10‐hexahydroacridine‐4,5(1H, 6H)‐dione (4l)

Yield: 0.44 g, 95%; mp 295–297°C; ^1^H NMR (400 MHz, DMSO‐d6) δ = 0.70 (6H, s, 2CH_3_), 0.89 (6H, s, 2CH_3_), 1.79 (2H, d, *J* = 9.7 Hz, CH_2_), 2.02 (2H, d, *J* = 9.8 Hz, CH_2_), 2.21–2.27 (4H, m, 2CH_2_), 5.15 (1H, s, CH), 7.20 (2H, d, *J* = 8.4 Hz, ArH), 7.27 (1H, t, *J* = 8.3 Hz, ArH), 7.44 (2H, t, *J* = 8.3 Hz, ArH), 7.56–7.65 (3H, m, ArH), 7.80 (1H, s, ArH), 7.80 (1H, d, *J* = 7.8 Hz, ArH); IR (KBr) 3073, 2955, 1640, 1582, 1269, 760 cm^−1^.

## 3 Results and Discussion

Micellar catalysis plays a significant role in green chemistry by offering a more sustainable alternative to traditional catalytic processes. The use of micellar systems enables reactions to occur in aqueous environments, thereby reducing the need for organic solvents that can be harmful to the environment. This can lead to lower energy consumption, decreased waste generation, and overall reduced environmental impact.

One of the key advantages of micellar catalysis is that it enables reactions to take place in an aqueous environment, which is often more environmentally friendly and cost‐effective than traditional organic solvent‐based reactions. The surfactant molecules in the micelles can also act as catalysts themselves, enhancing the rate of the chemical reaction.

### 3.1 Synthesis and Characterization of the Micellar Catalyst

**SCHEME 1:**
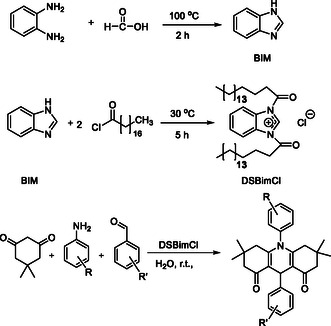
Preparation of DSBimCl for micellar‐promoted synthesis of *N*‐aryl‐1,8‐dioxo‐decahydroacridines.

The synthesis of new anionic surfactants by the acylation method using acyl chloride and a quaternary nitrogen group, such as benzimidazole, involves the reaction of a hydrophobic compound with an acyl chloride and a compound containing the quaternary nitrogen group to form an amide bond. This reaction results in the creation of an amphiphilic molecule that possesses both hydrophobic and anionic characteristics, which are crucial for surfactant function (Scheme 1).

The incorporation of a quaternary nitrogen group, such as benzimidazole, into the surfactant structure can impart specific properties, such as enhanced antimicrobial activity or increased solubility in certain environments. The acylation method allows for precise control over the structure and properties of the new anionic surfactant by adjusting reaction conditions and the selecting reactants.

**FIGURE 1:**
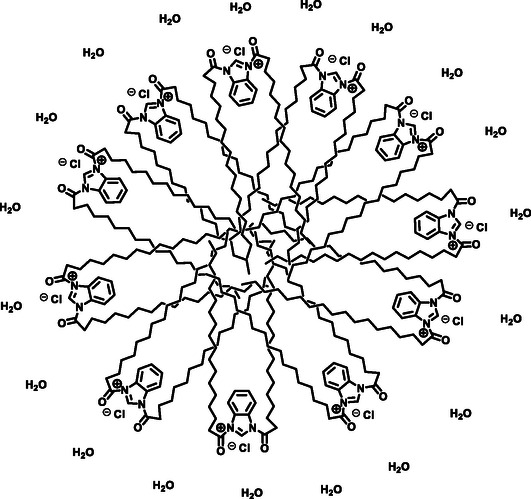
Micellar surfactant in water technology.

In the solubilization process, the hydrophobic tail of the anionic surfactant interacts with the organic substances, while the anionic head group remains in contact with the surrounding water molecules. This interaction helps to reduce the surface tension between the organic and aqueous phases, enabling better dispersion and solubility of the organic substances in water (Figure 1).

**FIGURE 2:**
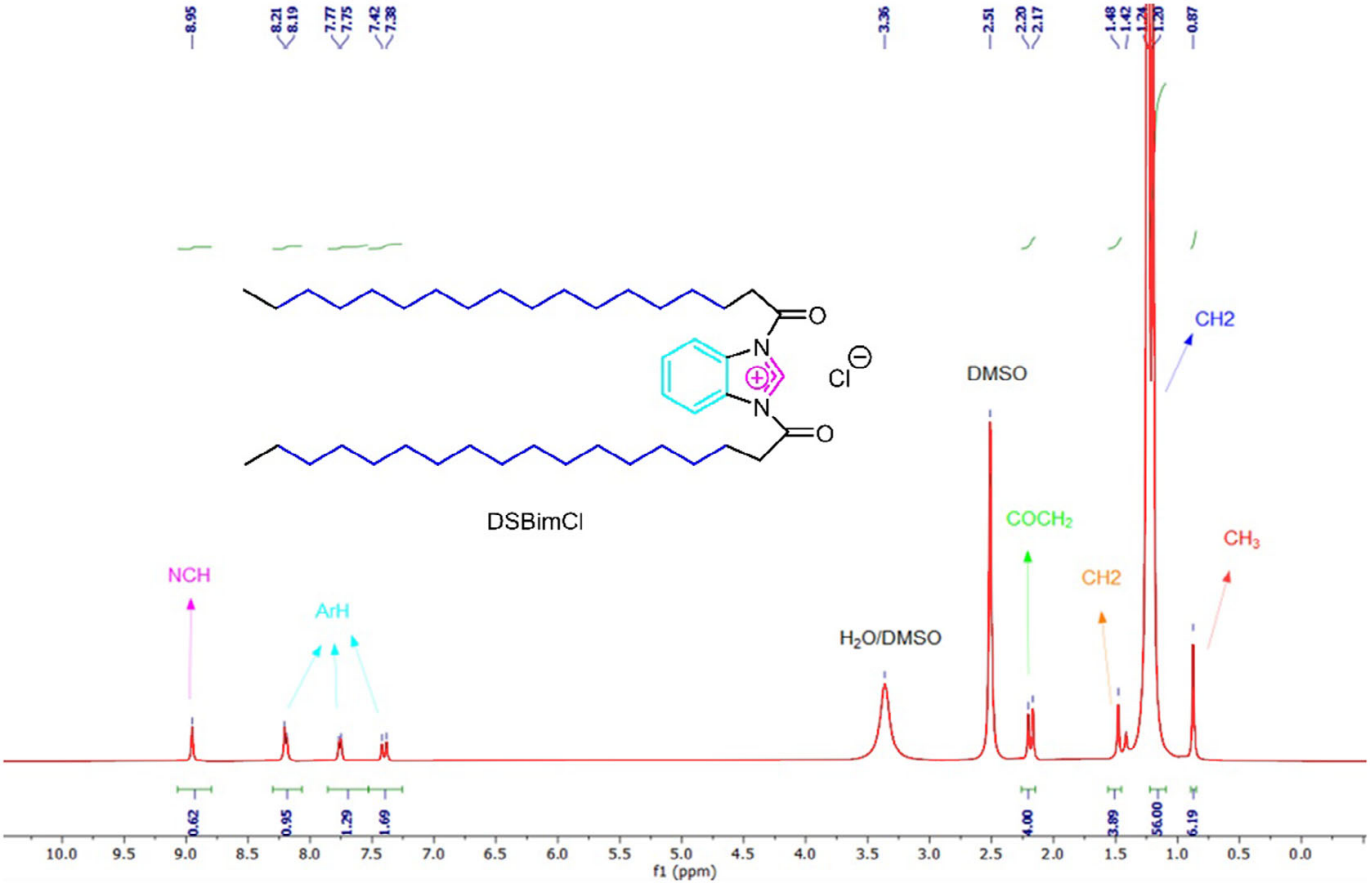
The ^1^H NMR of the DSBimCl surfactant.

The new catalyst demonstrated improved performance as a surfactant compared to traditional options, due to its lower CMC, higher polarity, and enhanced self‐assembly capabilities. To create this new anionic surfactant, we designed BIM, which incorporates two quaternary nitrogen atoms and an acyl group attached to a hydrophobic tail. This compound had previously been synthesized for other applications, and we prepared it as a white solid with a melting point of 62–65°C. Its structure was confirmed through ^1^H NMR, ^13^C NMR, and FT‐IR analyses. To further investigate and validate the structure of the new surfactant, we examined the spectra obtained from FT‐IR and the magnetic resonance analyses of hydrogen and carbon nuclei (^1^H NMR and ^13^C NMR). The ^1^H NMR spectrum of the surfactant confirms its molecular structure through characteristic signals (Figure 2).

**FIGURE 3:**
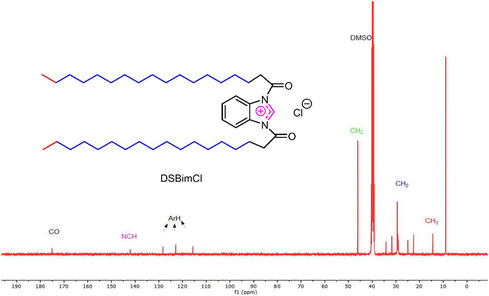
The ^13^C NMR of the DSBimCl surfactant.

The proton NMR spectrum reveals a peak at 0.87 ppm (CH_3_) and a broad signal at 1.20–1.24 ppm (CH_2_), with integrals matching the expected hydrogen count in the compound's aliphatic chains. Additionally, the NMR signals at 1.48 ppm (CH_2_), 2.17–2.20 ppm (methylene near carbonyl), and 7.38–8.21 ppm (aromatic protons) correspond to the expected hydrogen environments in the compound. The ^1^H NMR also shows a signal at ∼ 9 ppm, consistent with the imidazole proton. In the ^13^C NMR spectrum, signals at 175 ppm (carbonyl), 145 ppm (imidazole), 115–130 ppm (aromatic), 47 ppm (α carbon to carbonyl), and 15–25 ppm (aliphatic chains) confirm the expected structure (Figure 3).

**FIGURE 4:**
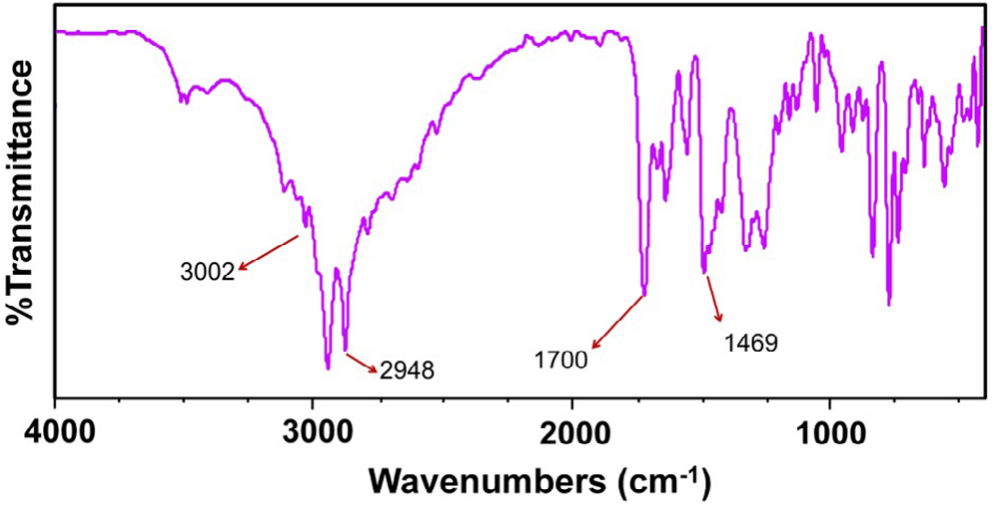
The FT‐IR of the DSBimCl surfactant.

In the FT‐IR spectrum (Figure 4), the signal at 1700 cm^−1^ corresponds to the carbonyl amide group. Additionally the peaks at 1469 and 1617 cm^−1^ correspond to the aromatic ring, while the regions from 2848 to 3002 cm^−1^ relate to the stretching CH bonds of aliphatic chains.

The CMC is a key parameter that indicates the concentration at which surfactant molecules start to form micelles. Determining the CMC value is crucial for understanding surfactant aggregation behavior, which in turn governs their interfacial activity, solubilization capacity, and colloidal stability. In practical applications such as drug delivery and emulsification, knowing the CMC values helps optimize the use of surfactants and formulation. Additionally, CMC values indicate the purity of surfactants and their interactions with other substances. To investigate the aggregation behavior of the DSBimCl surfactant, its CMC was determined using the conductometric method at 25°C. For this purpose, a series of aqueous solutions with varying concentrations of the DSBimCl was prepared, and their specific conductivity was measured using a calibrated conductometer. The plot of conductivity versus concentration exhibited two linear regions with different slopes, and the intersection point of these lines was taken as the CMC value. The CMC of the synthesized surfactant was found to be 2.6 mM, indicating a favorable tendency of the surfactant molecules to self‐assemble and form micellar structures in aqueous media.

**FIGURE 5:**
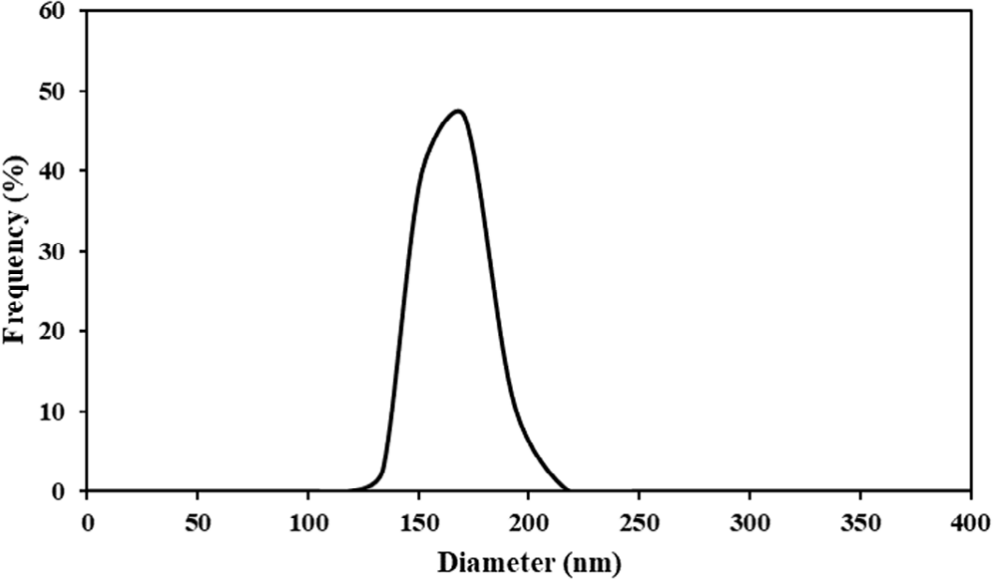
Dynamic light scattering (DLS) analysis of the DSBimCl surfactant.

Dynamic light scattering analysis of the surfactant DSBimCl revealed an average micelle size of 155 nm in the aqueous reaction medium, as shown in Figure 5. This micelle size enhances the catalytic activity of DSBimCl in the multicomponent synthesis of acridine derivatives, as larger micelles provide a more effective hydrophobic microenvironment, improving reactant solubility and reaction efficiency.

### 3.2 Description of Catalytic Activity of the Synthesized Micelle

In the preliminary stage of our research, we chose the synthesis of the compound 1,8‐dioxodecahydroacridines via the reaction of dimedone, 4‐nitrobenzaldehyde, and an aniline as a model reaction. This approach aimed to assess the efficiency of DSBimCl as a catalyst and determine the best reaction conditions. Table 1 provides a summary of the optimization experiments conducted.

**TABLE 1:** Synthesis of compound *N*‐aryl‐1,8‐dioxo‐decahydroacridines in the presence of DSBimCl as catalyst in different reaction conditions.^a^

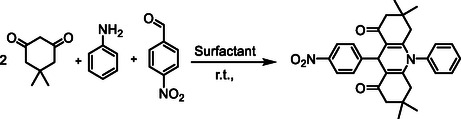				
Entry	Surfactant (g)	Solvents	Time (min)	Yield (%)^b^
1	—	H_2_O	240	9
2	0.1	H_2_O	180	64
3	0.15	H_2_O	120	70
4	0.20	H_2_O	30	96
5	0.25	H_2_O	32	85
6	0.20	MeOH	35	80
7	0.20	CH_3_CN	60	70
8	0.20	EtOH	35	75

a In a reaction vessel a mixture of dimedone (2 mmol),4‐nitro benzaldehyde (1 mmol), and aniline (1 mmol) were performed under room temperature and conditions of any selected entry;

b Isolated yields.

The first experiment performed without a catalyst produced only 9% of the desired product after 4 h (entry 1). Following this, the reaction was conducted using various amounts of the catalyst and in different solvents. The findings suggest that the reaction's efficiency is mainly determined by both the quantity of DSBimCl and the selected solvent. The best results were obtained with 0.2 g of DSBimCl (entry 4). To illustrate the effect of the solvent, the same model reaction was performed using different solvents, such as EtOH, MeOH, H_2_O, and CH_3_CN, with 0.2 g of the catalyst (entries 6–8). The results revealed that water as the solvent resulted in a higher yield and a considerably shorter reaction time when compared to traditional methods. To emphasize the benefits of this methodology, the outcomes were compared with those from other reported techniques for synthesizing 1,8‐dioxodecahydroacridines, as detailed in Table 2.

**TABLE 2:** Comparison of efficiency of various surfactants in the synthesis of 1,8‐dioxodecahydroacridines.^a^

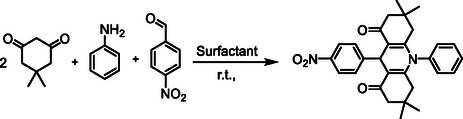			
Entry	Surfactant (0.2 g)	Time (min)	Yield b %
1	SDS	150	85
2	CTAB	120	65
3	DSBimCl	30	96
4	MTPPB	180	40
5	Tween 20	240	70

a In a reaction vessel a mixture of dimedone (2 mmol),4‐nitro benzaldehyde (1 mmol), and aniline (1 mmol) were performed under room temperature and selected surfactant;

b Isolated yields.

As can be seen, different neutral, anionic, and cationic surfactants were employed to evaluate their effects on product formation. Our method notably decreases reaction times while achieving high yields of the desired products.

Mechanically, the reaction involves a one‐pot, three‐component coupling of an anilines, 4‐nitro benzaldehyde, and dimedone using DSBimCl as a novel, efficient, and nontoxic catalyst. All reactions yielded good to excellent results within a short time frame. In a thorough investigation of the synthetic process, various electronically distinct aryl aldehydes were reacted with dimedone and anilines under the same conditions, and all these substrates demonstrated similar responsiveness. Notably, several heteroaryl aldehydes also participated in the reaction, though at a slower rate. A summary of representative results can be found in Table 3.

**TABLE 3:** DSBimCl facilitated the production of various 1,8‐dioxodecahydroacridines derivatives in water.

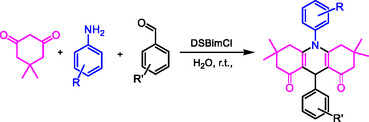			
4**a**‐4**l**: Heating^a^ Time, Yield (%) m.p°C Reference m.pºC (Found)			
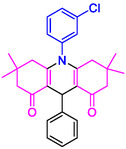 **4a**: 32 min, 71% m.pºC [ref]: 177–180 [39] m.pºC (Fnd): 179–181	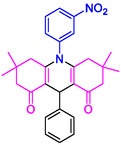 **4b**: 31 min, 95% m.pºC [ref]: new m.pºC (Fnd): 205–208	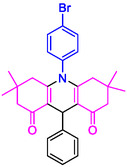 **4c**: 29 min, 95% m.pºC [ref]: 270–272 [61] m.pºC (Fnd): 302–303	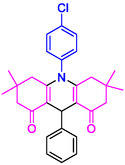 **4d**: 28 min, 98% m.pºC [ref]: 301–303 [62] m.pºC (Fnd): 219–221
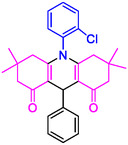 4e: 29 min, 95% m.pºC [ref]: 188–190 [63] m.pºC (Fnd): 187–189	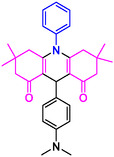 4f: 33 min, 90% m.pºC [ref]: 165–167 [64] m.pºC (Fnd): 169–171	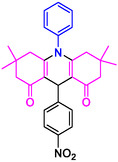 4g: 30 min, 96% m.pºC [ref]: 281–282 [65] m.pºC (Fnd): 280–283	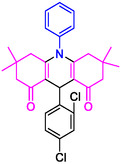 4hr: 28 min, 93% m.pºC [ref]: new m.pºC (Fnd): 288–290
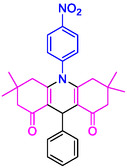 4i: 36 min, 91% m.pºC [ref]: 248–250 [66] m.pºC (Fnd): 248–250	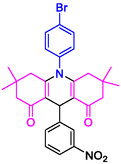 4j: 30 min, 96% m.pºC [ref]: 295–296 [67] m.pºC (Fnd): 291–294	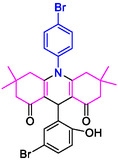 4k: 32 min, 96% m.pºC [ref]: new m.pºC (Fnd): 207–210	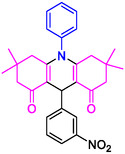 4l: 30 min, 95% m.pºC [ref]: 297–299 [68] m.pºC (Fnd): 295–297

a Thermal circumstances: aromatic amine (1 mmol), benzaldehyde (1 mmol), dimedone (2 mmol), H_2_O (10 mL), T, room temperature; DSBimCl (0.2 g).

The reaction of 1,8‐dioxodecahydroacridines with benzaldehyde, aniline, and dimedone typically leads to the formation of complex polycyclic compounds through a sequence of condensation and cyclization reactions. Aniline reacts with benzaldehyde to form an imine. The lone pair of electrons on the nitrogen atom of the amine attacks the carbonyl carbon of benzaldehyde, resulting in the formation of the imine while releasing water. The imine formed in the previous step can act as a nucleophile. The α‐carbon of the 1,8‐dioxodecahydroacridine can be activated due to the electron‐withdrawing nature of the adjacent carbonyl groups, allowing the imine to attack this carbon center. This step can lead to the formation of a new carbon‐to‐carbon bond and an intermediate that has both an imine and two carbonyls. The intermediate will undergo cyclization, with dimedone (5,5‐dimethyl‐1,3‐cyclohexanedione) acting as a nucleophile. The enol form of dimedone can attack the carbonyl carbon of the intermediate formed in the previous step.

This leads to the formation of a new ring and the establishment of a tetrahydroacridine structure with additional substituents from dimedone. Further cyclization may occur, where the nitrogen in the imine can assist in stabilizing the reaction intermediate through resonance or facilitating proton transfers. Dehydration might happen during rearrangement, further stabilizing the cyclic structure. The final product is a complex polycyclic compound that contains features from both the 1,8‐dioxodecahydroacridine and the dimedone, as well as the benzaldehyde and aniline inputs.

**FIGURE 6:**
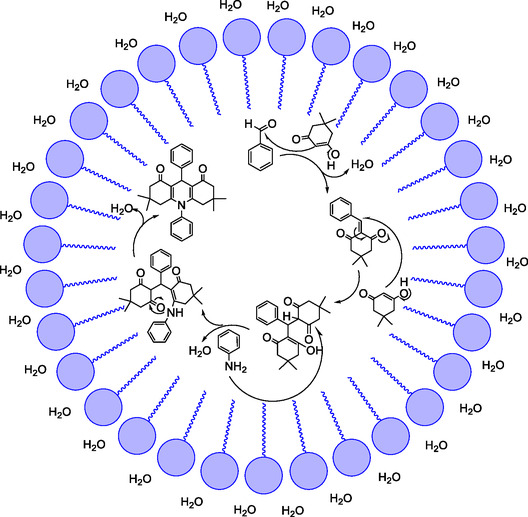
Schematic diagram representing the role of DSBimCl.

Overall, the mechanism involves the formation of an imine, nucleophilic attack by an enolate of dimedone, cyclization to form a polycyclic structure, and dehydration processes (Figure 6).

The synthesis of new anionic surfactants using the acylation method with a quaternary nitrogen group provides a versatile approach to developing surfactants with suitable properties for specific applications across various industries. In summary, we have successfully developed a simple and environmentally friendly method for synthesizing 1,8‐dioxodecahydroacridines with a high yield using anionic micelles (DSBimCl). This approach offers several advantages, including ease of operation, fast reaction time, and low catalyst loading.

## 4 Conclusion

Micellar catalysis, through the innovative use of carefully designed nanoreactors, has become an exceptionally effective method for water‐based reactions, providing a sustainable alternative to traditional organic solvents that generate waste. The results of this study demonstrate that surfactants act as powerful agents or catalysts, facilitating and accelerating organic reactions in aqueous environments.

We have developed a simple, mild, and ecofriendly synthetic protocol for the preparation of 1,8‐dioxodecahydroacridines. Our method incorporates DSBimCl for organic transformation, marking its first reported application in this context. Utilizing water as a solvent not only proves to be cost‐effective but also significantly enhances reactivity and selectivity. Our technique utilizes DSBimCl as a catalyst at room temperature, enabling rapid reaction times while eliminating the need for hazardous organic reagents and solvents.

## Supporting Information

Some characteristics of the catalyst and products are described in the ESI available through: stl.publisher.ingentaconnect.com/content/stl/jcr/supp‐data. Additional supporting information can be found online in the Supporting Information section.

## Conflicts of Interest

The authors declare no conflicts of interest.

## Supporting information

Supplementary Material

## Data Availability

The data that support the findings of this study are available in the supplementary material of this article.
