# Supplementary material for: Surfactant Derived From Stearoyl Chloride and Benzimidazole for Micellar‐Promoted Synthesis of N‐Aryl‐1,8‐dioxo‐decahydroacridines in Water at Room Temperature
Source: ChemistryOpen. 2026 Jan 28;15(1):e202500600. doi: 10.1002/open.202500600 (PMC12848590; doi:10.1002/open.202500600)

Supporting information

Surfactant Derived from Stearoyl Chloride and Benzimidazole for Micellar-Promoted Synthesis of *N*-Aryl-1,8-dioxo-decahydroacridines in Water at Room Temperature

Saeedeh Asadian, Mohsen Moradian, Javad Safari*

Department of Organic Chemistry, Faculty of Chemistry, University of Kashan, Kashan, P.O. Box 87317-51167, I. R.Iran

10-(3-chlorophenyl)-3,3,6,6-tetramethyl-9-phenyl-2,3,7,8,9,10-hexahydroacridine-4,5(1H,6H)-dione (4a):

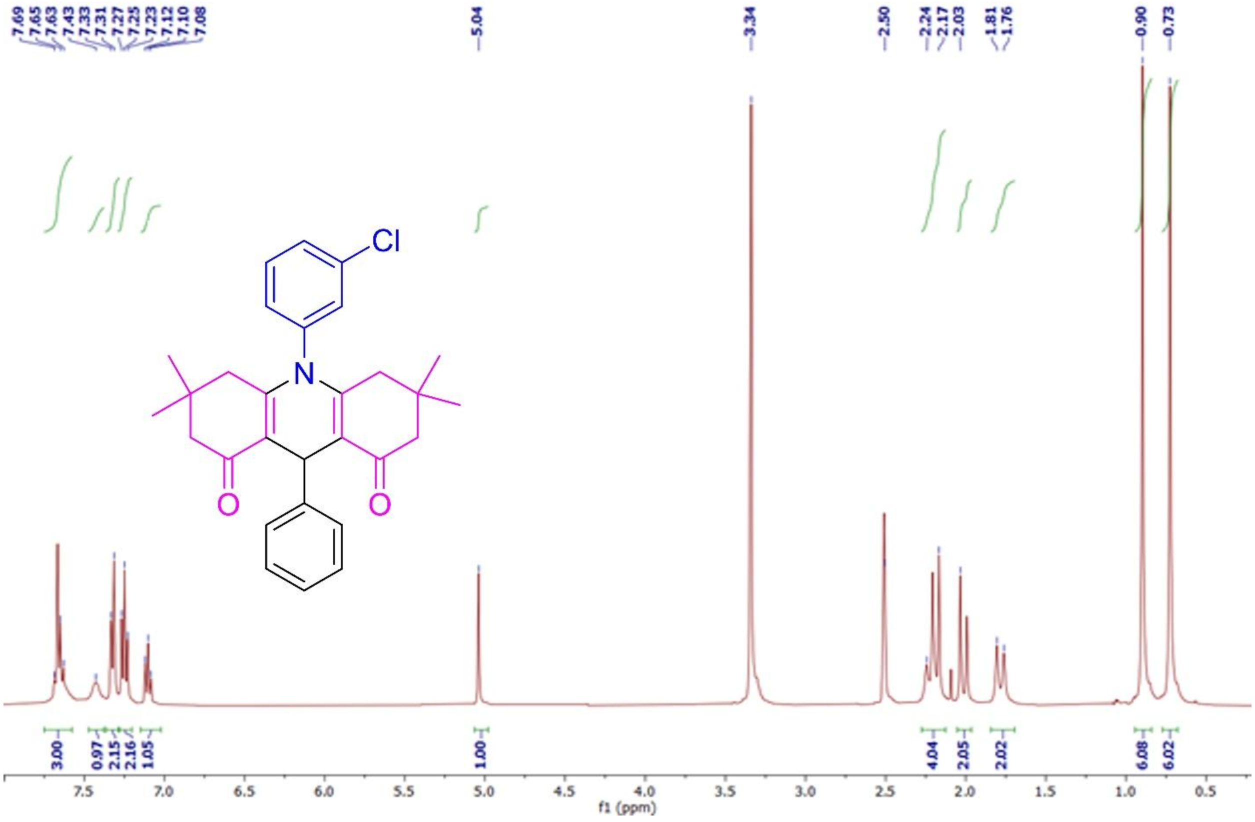

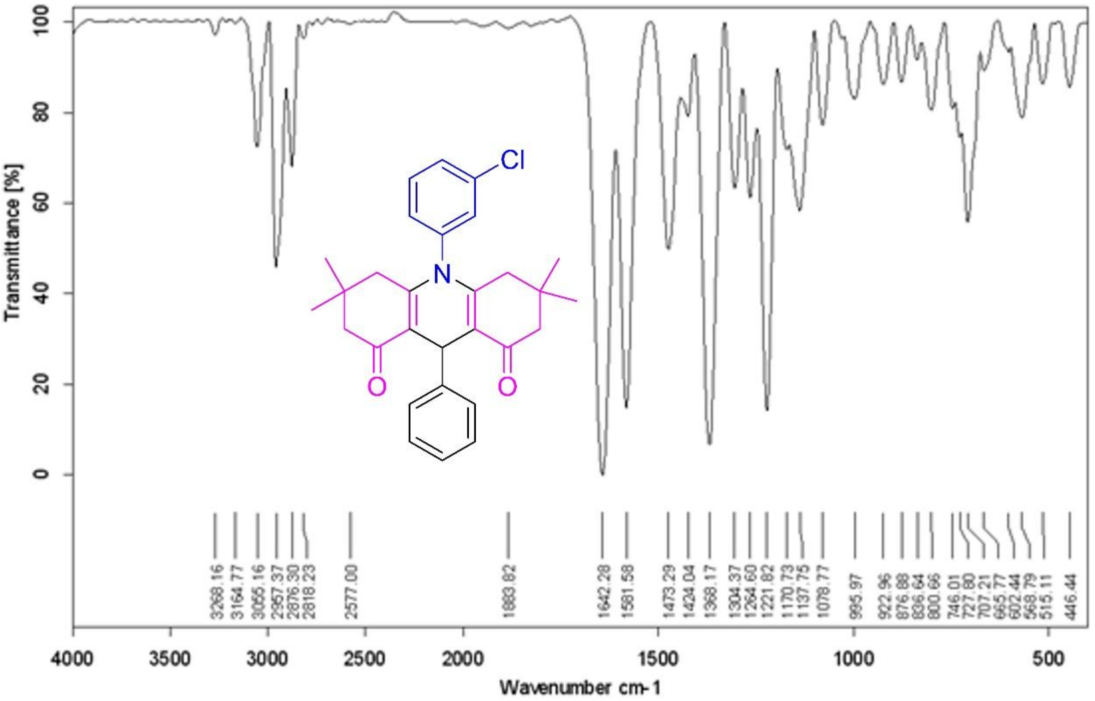

10-(3-nitrophenyl)-3,3,6,6-tetramethy l-9-phenyl-2,3,7,8,9,10-hexahydroacridine-4,5(1H,6H)-dione (4b):

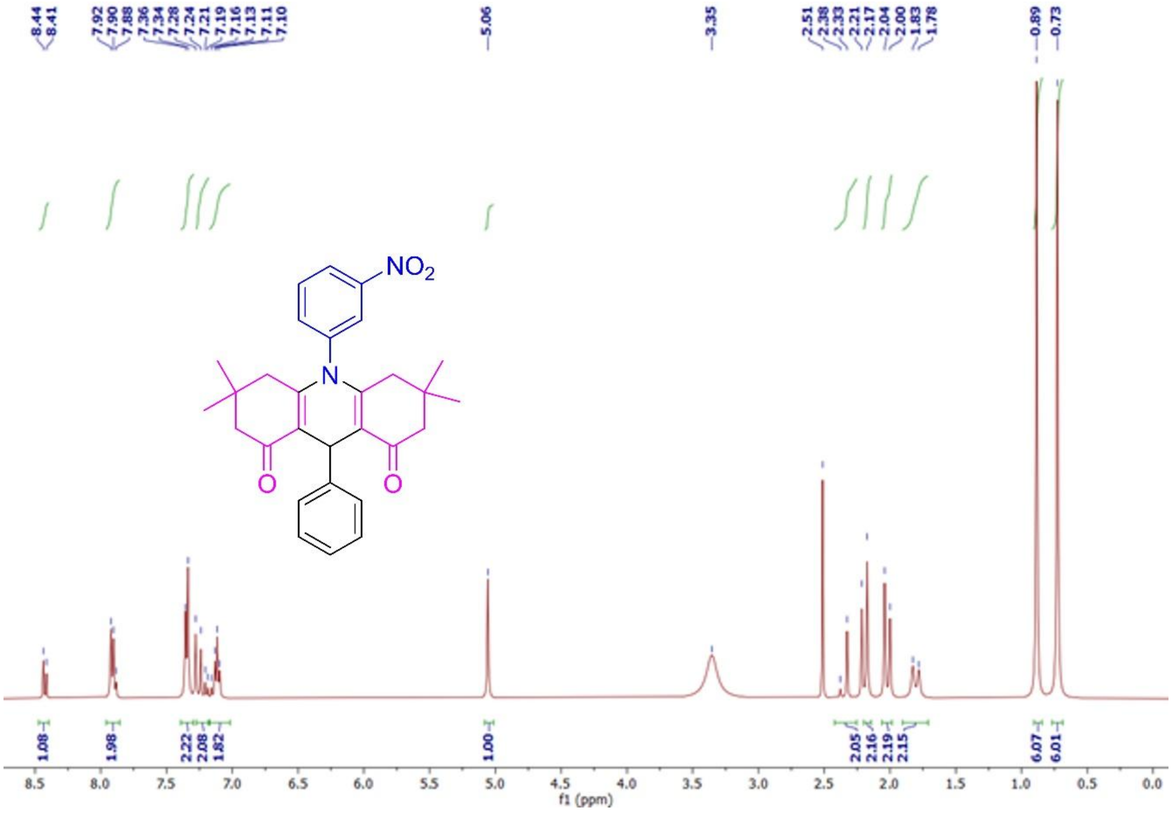

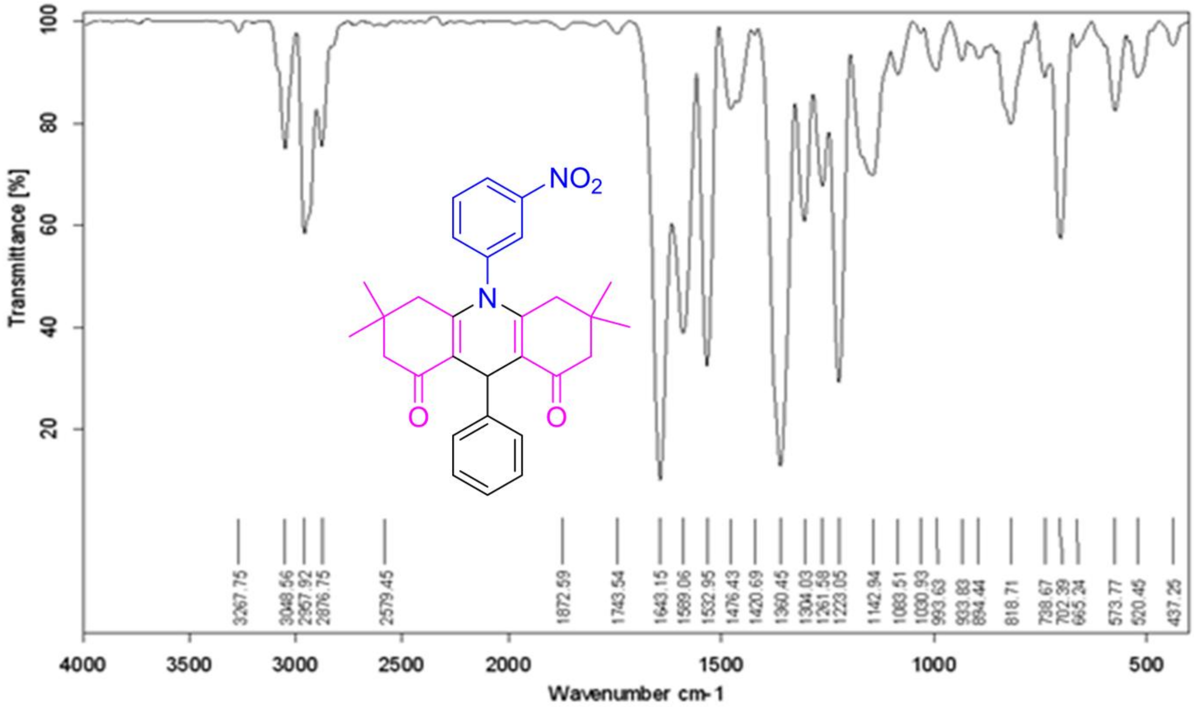

10-(4-bromophenyl)-3,3,6,6-tetramethyl-9-phenyl-2,3,7,8,9,10-hexahydroacridine-4,5(1H,6H)-dione (4c)

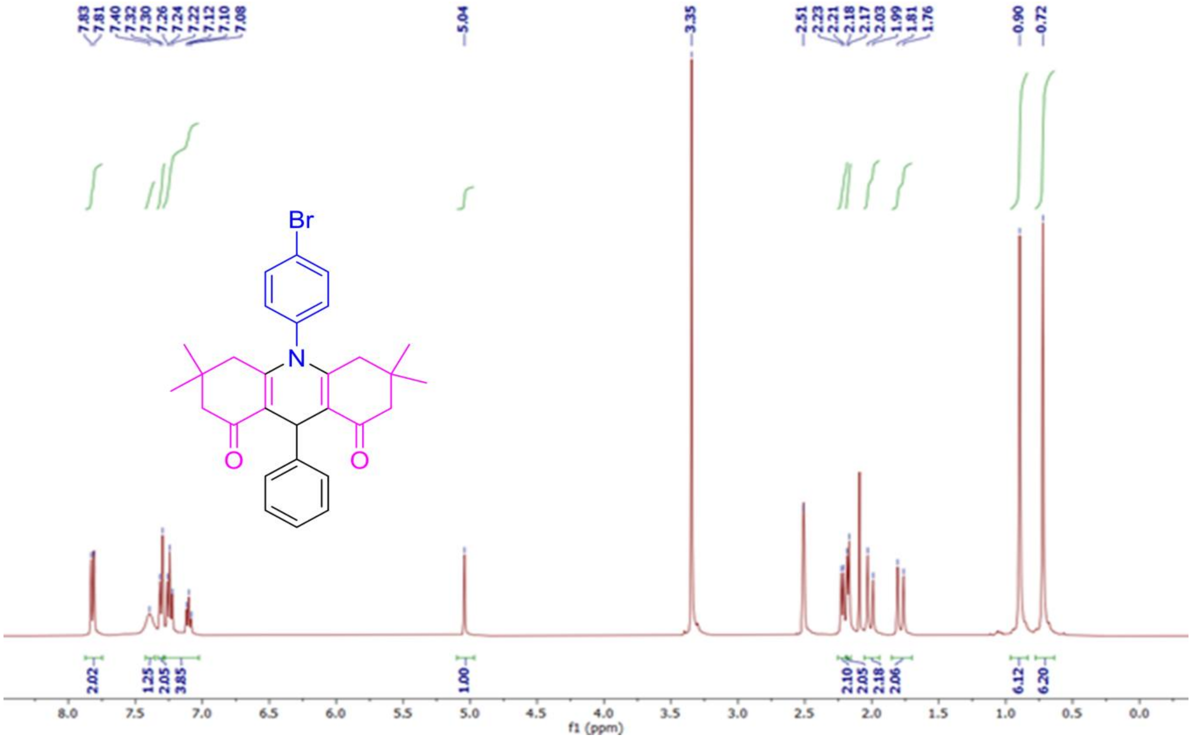

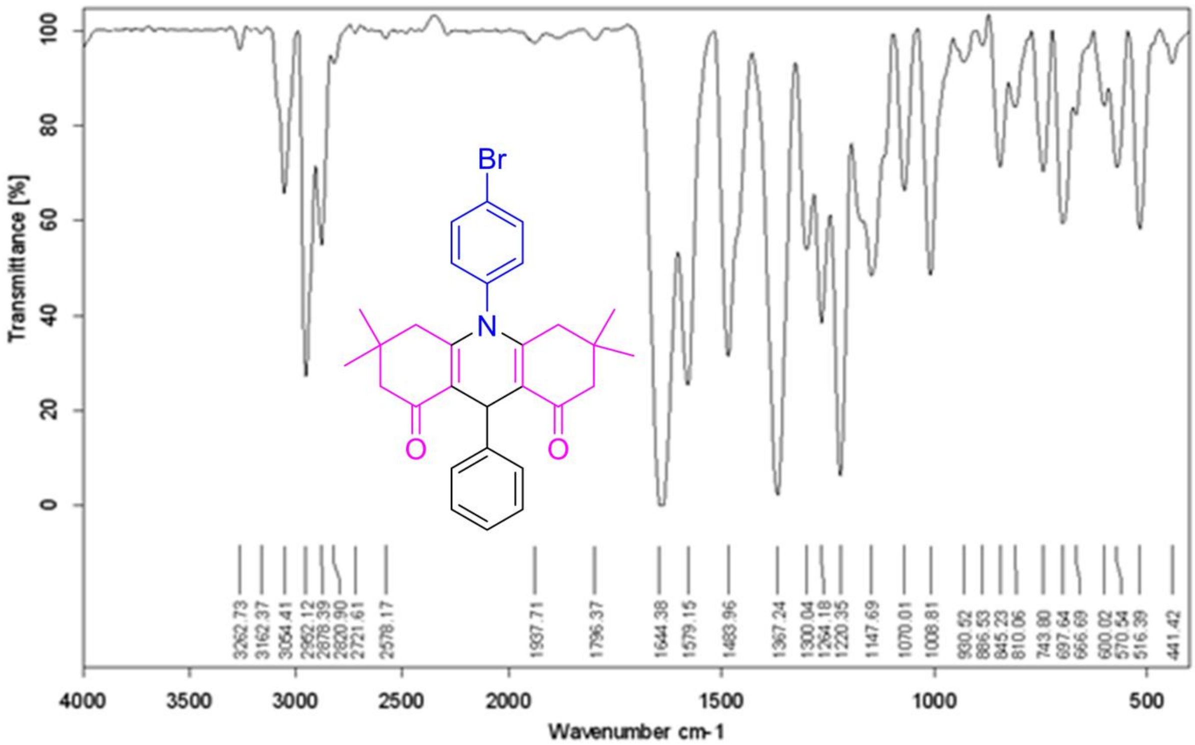

10-(4-chlorophenyl)-3,3,6,6-tetramethyl-9-phenyl-2,3,7,8,9,10-hexahydroacridine-4,5(1H,6H)-dione (4d):

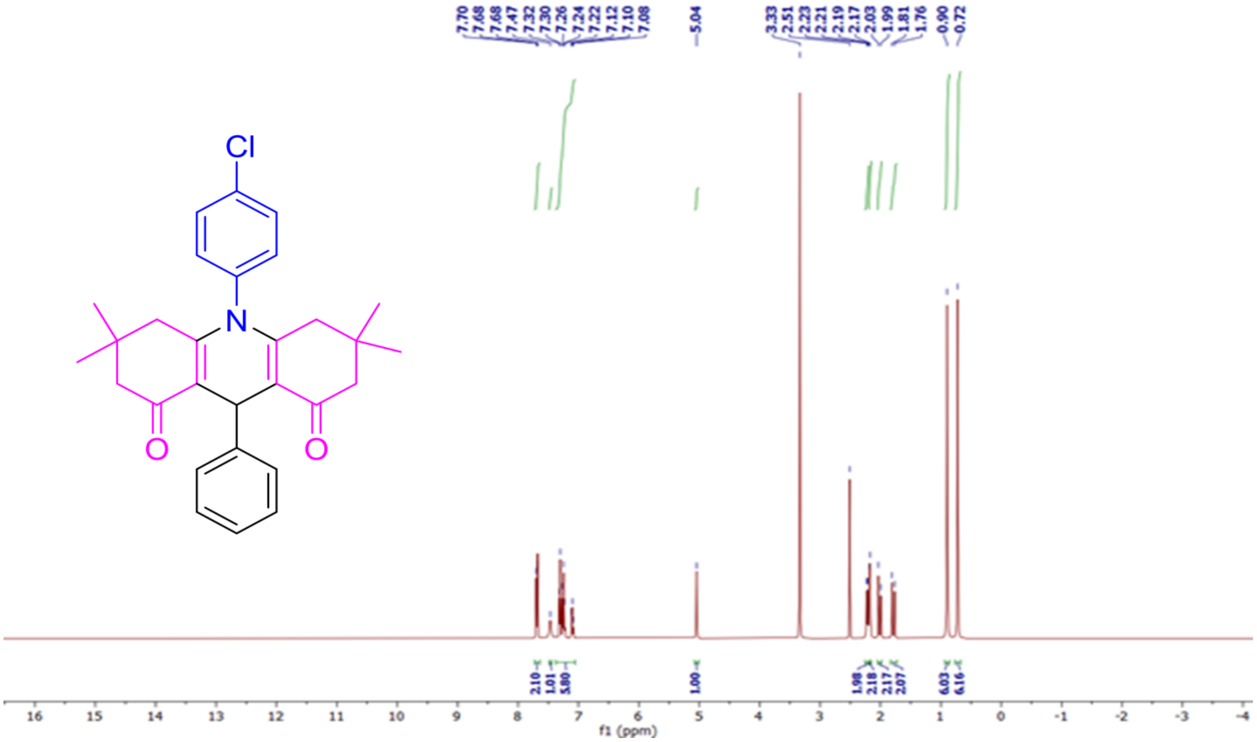

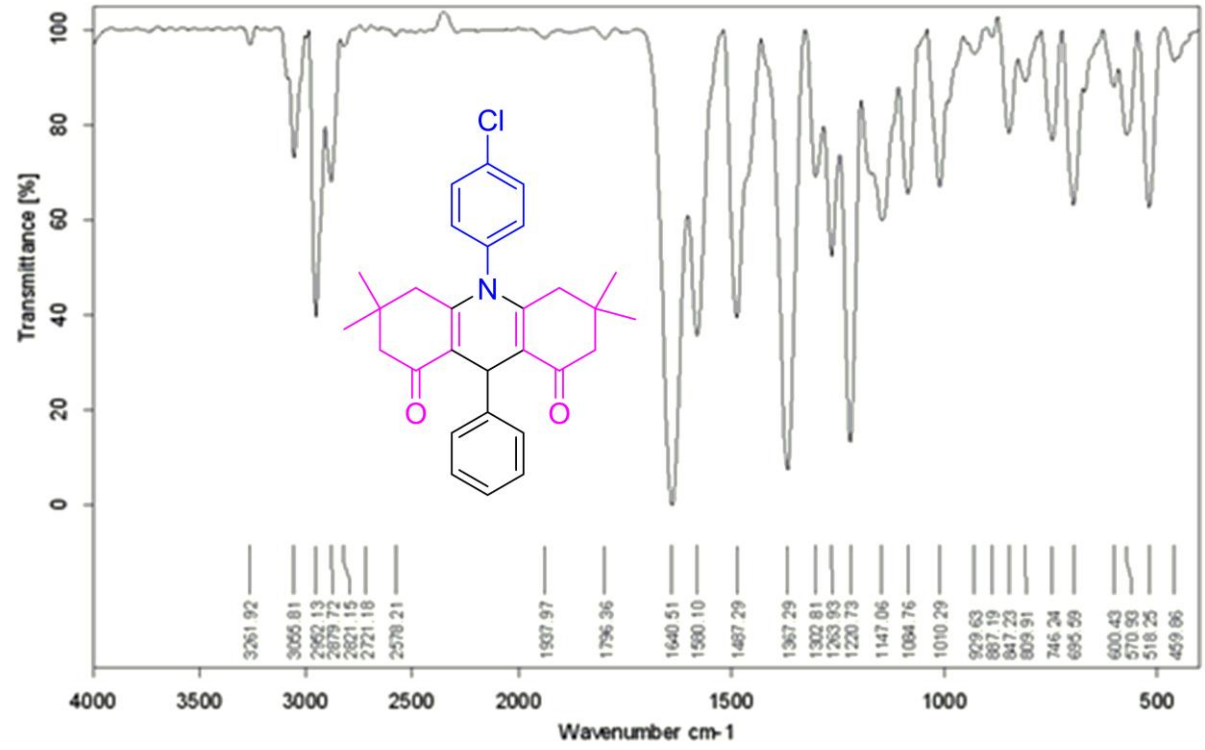

10-(2-chlorophenyl)-3,3,6,6-tetramethyl-9-phenyl-2,3,7,8,9,10-hexahydroacridine-4,5(1H,6H)-dione (4e):

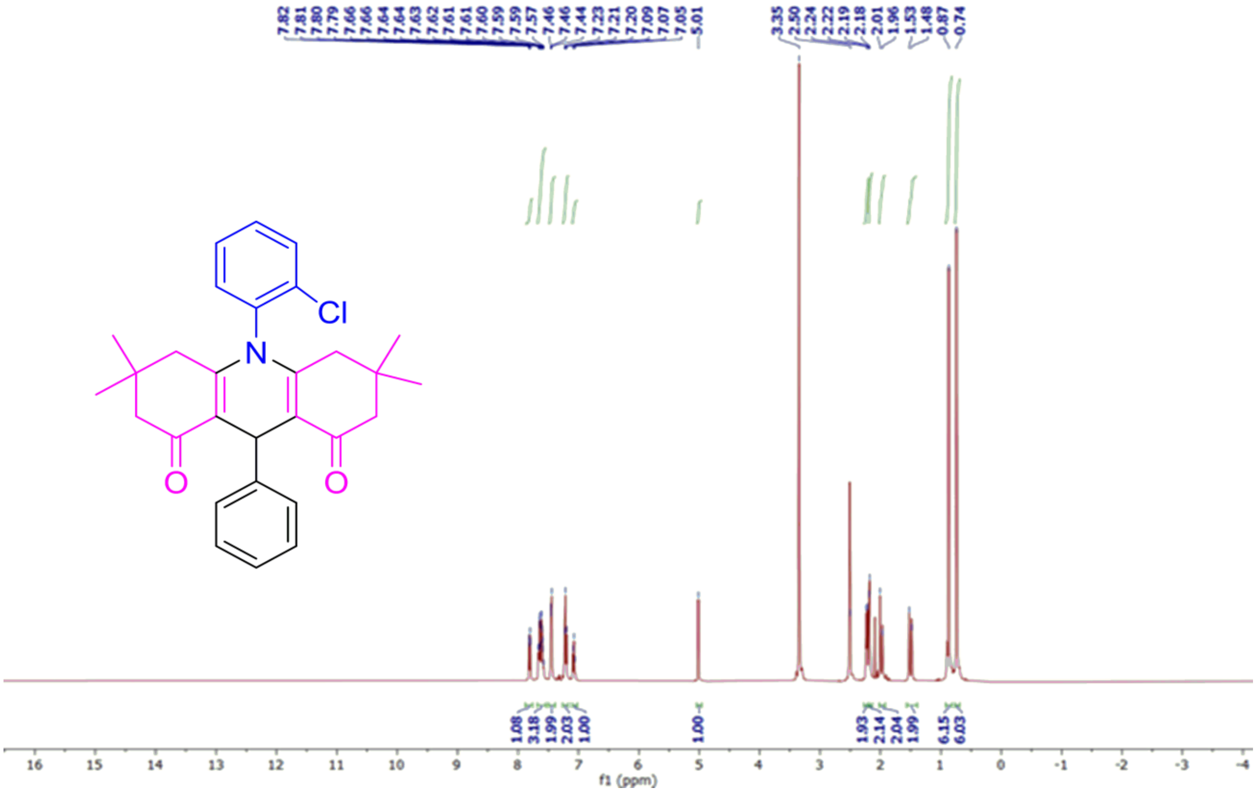

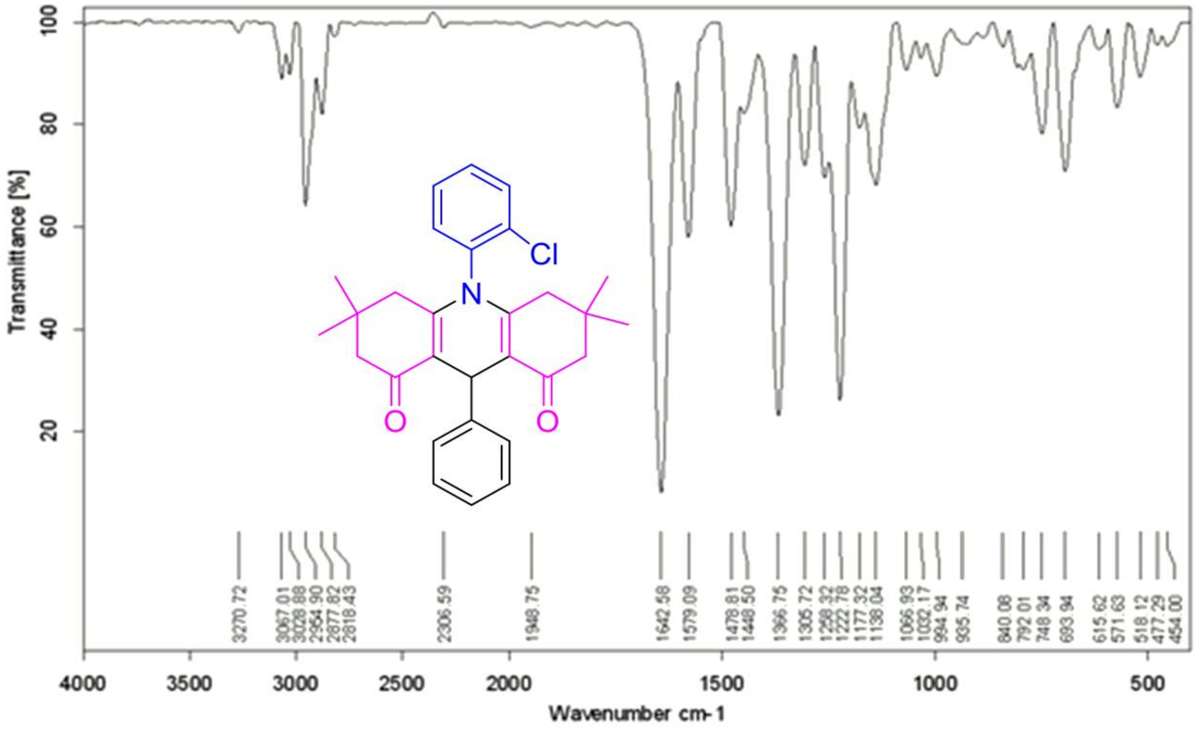

9-(4-(dimethylamino) phenyl)-3,3,6,6-tetramethyl-10-phenyl-2,3,7,8,9,10-hexahydroacridine-4,5(1H,6H)-dione (4f):

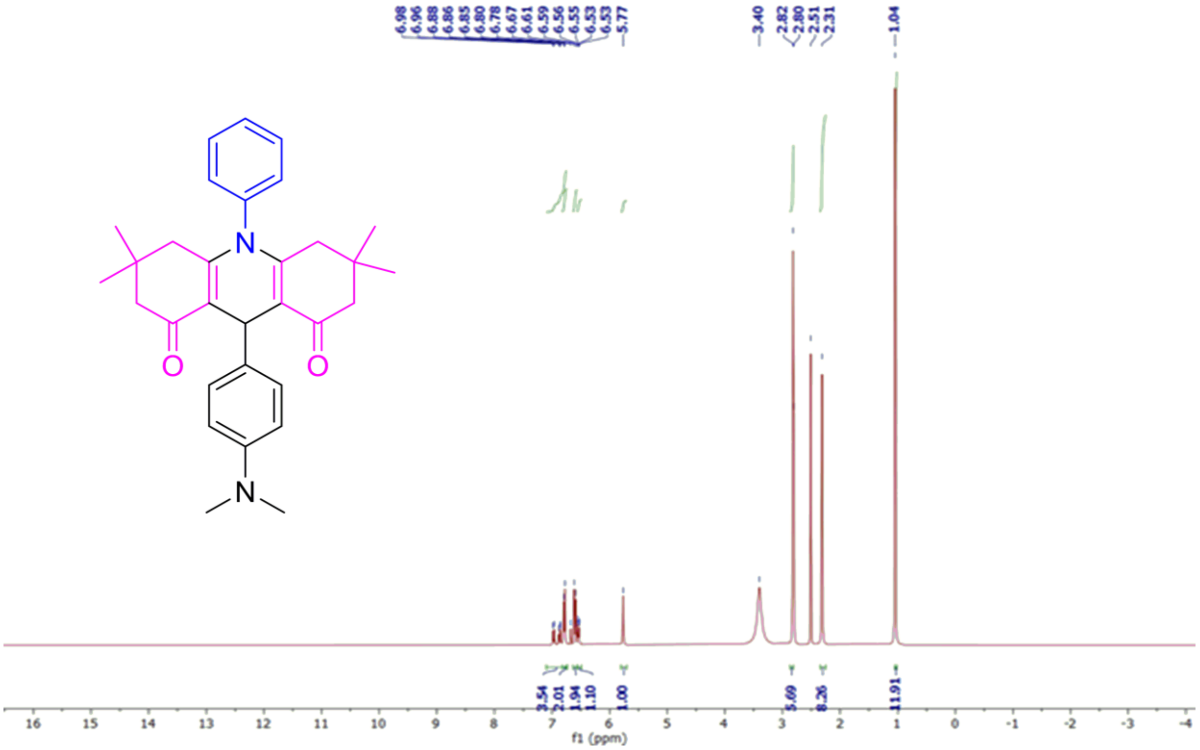

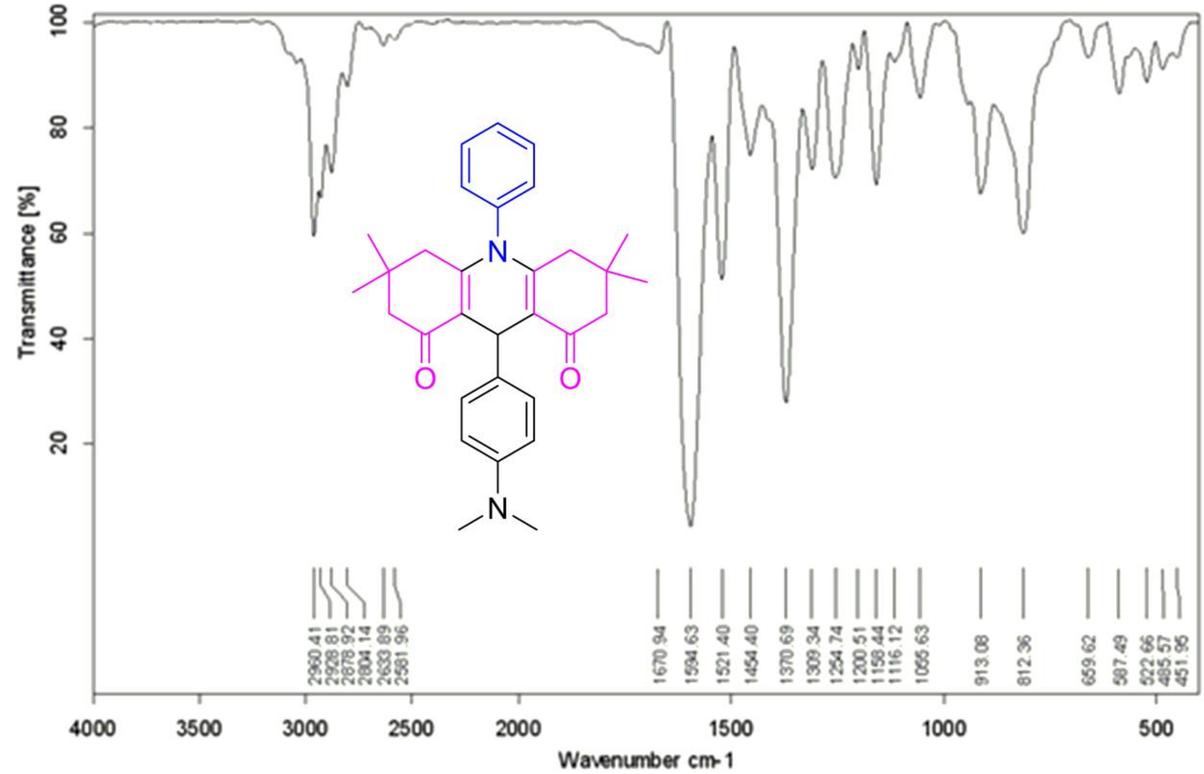

9-(4-nitrophenyl)-3,3,6,6-tetramethyl-10-phenyl-2,3,7,8,9,10-hexahydroacridine-4,5(1H,6H)-dione (4g):

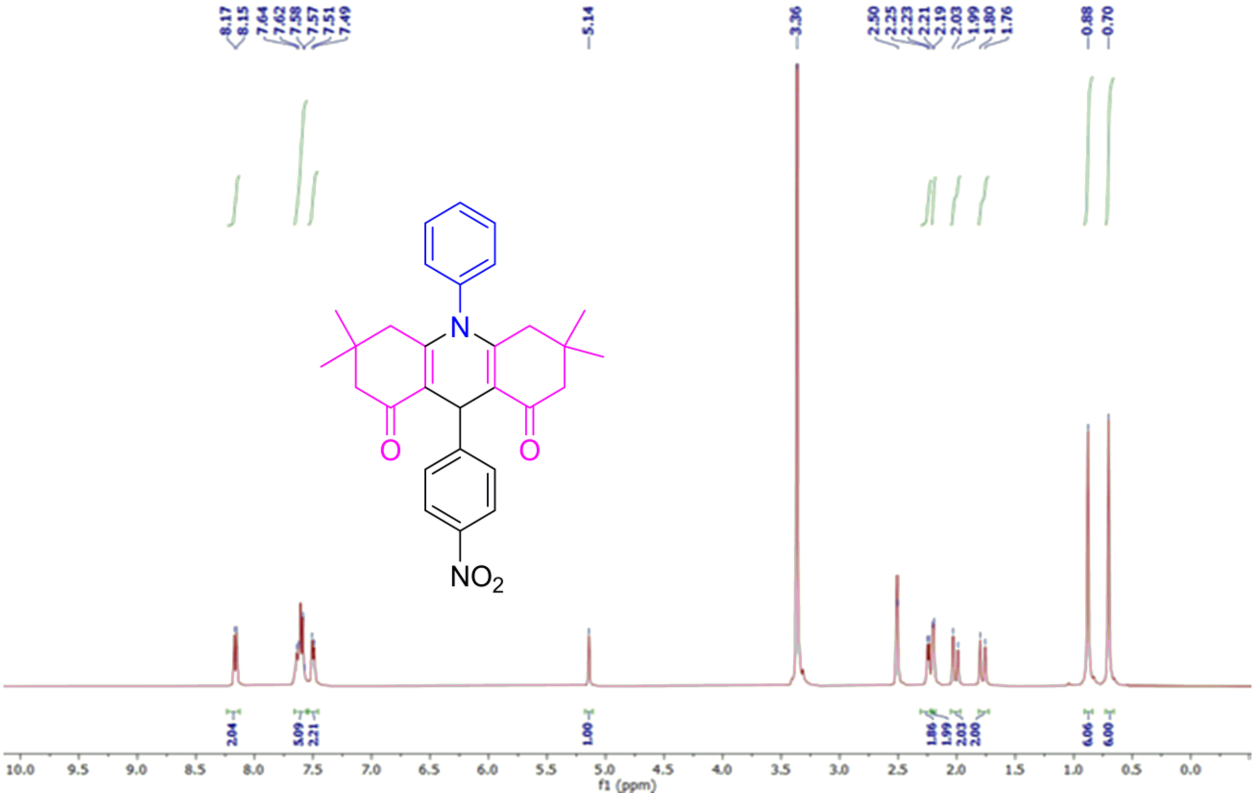

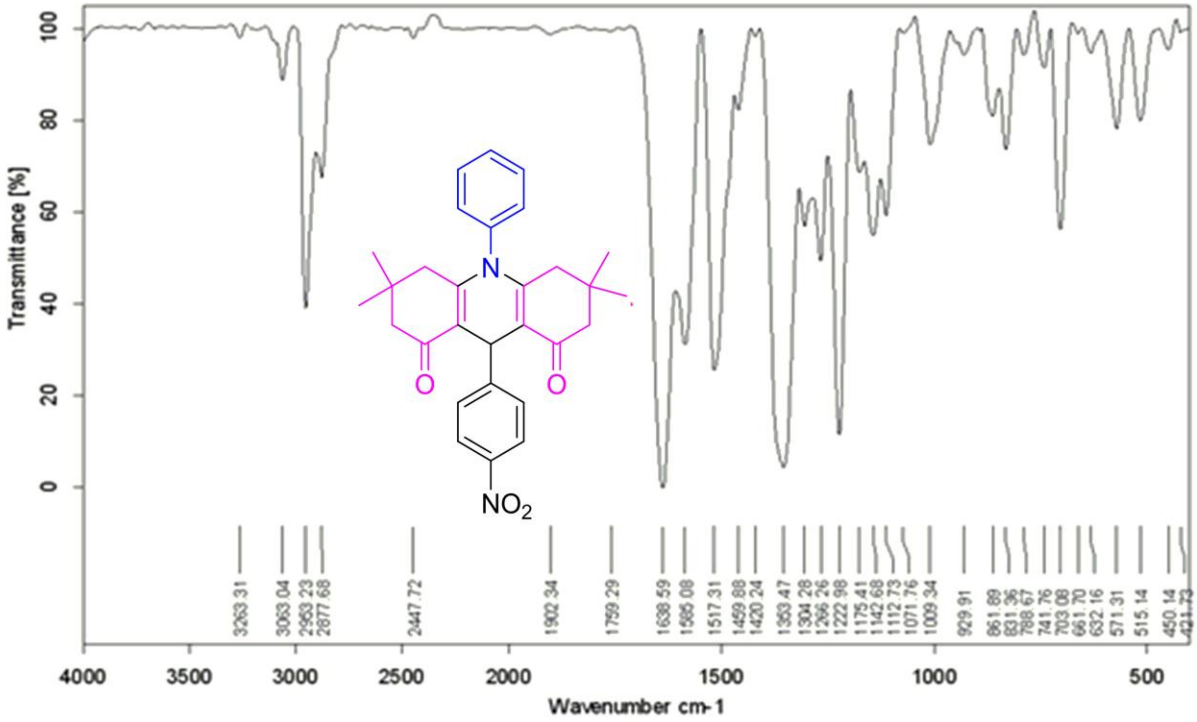

9-(2,4-dichlorophenyl)-3,3,6,6-tetramethyl-10-phenyl-2,3,7,8,9,10-hexahydroacridine-4,5(1H,6H)-dione (4h):

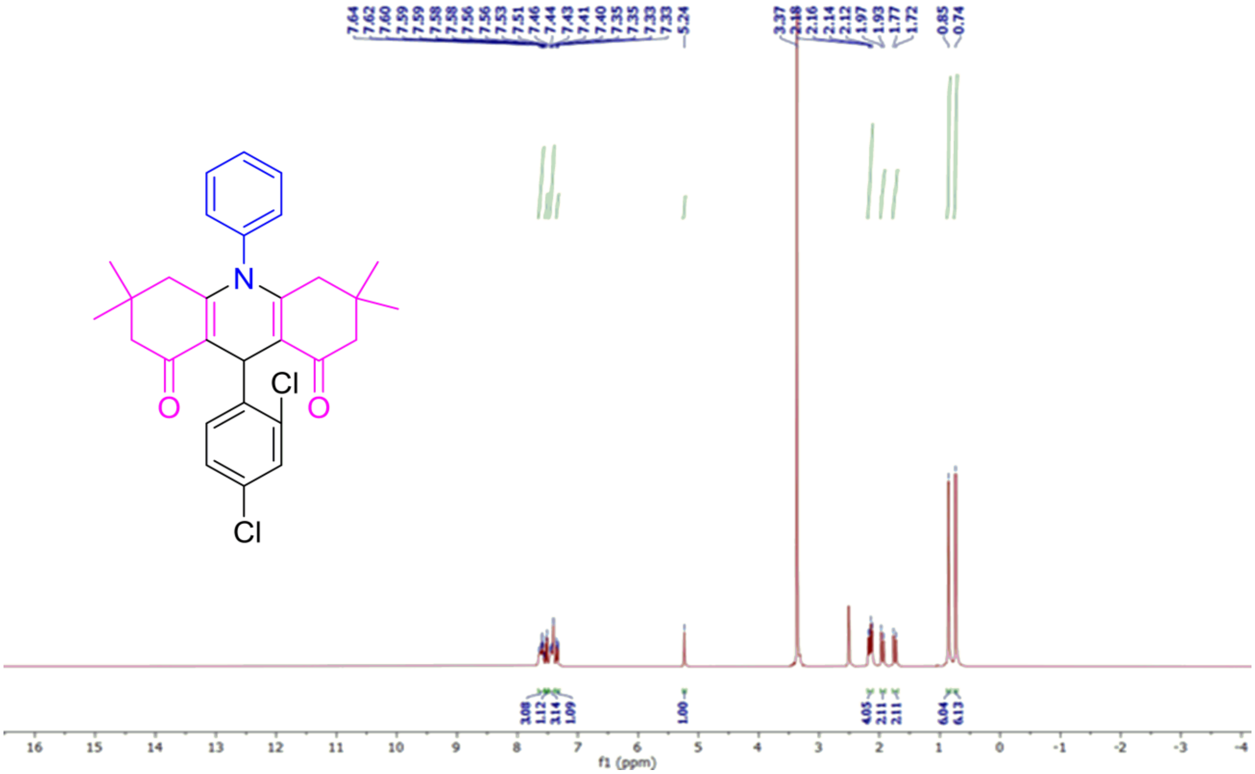

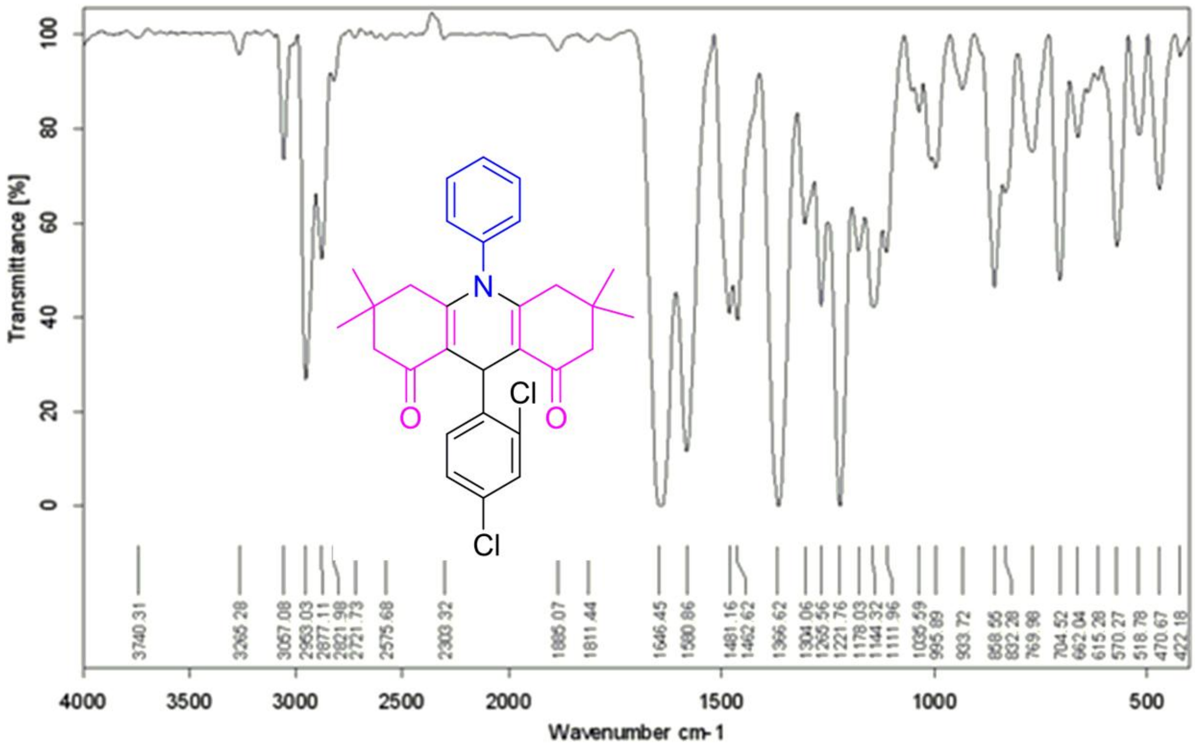

10-(4-nitrophenyl)-3,3,6,6-tetramethyl-9-phenyl-2,3,7,8,9,10-hexahydroacridine-4,5(1H,6H)-dione (4i):

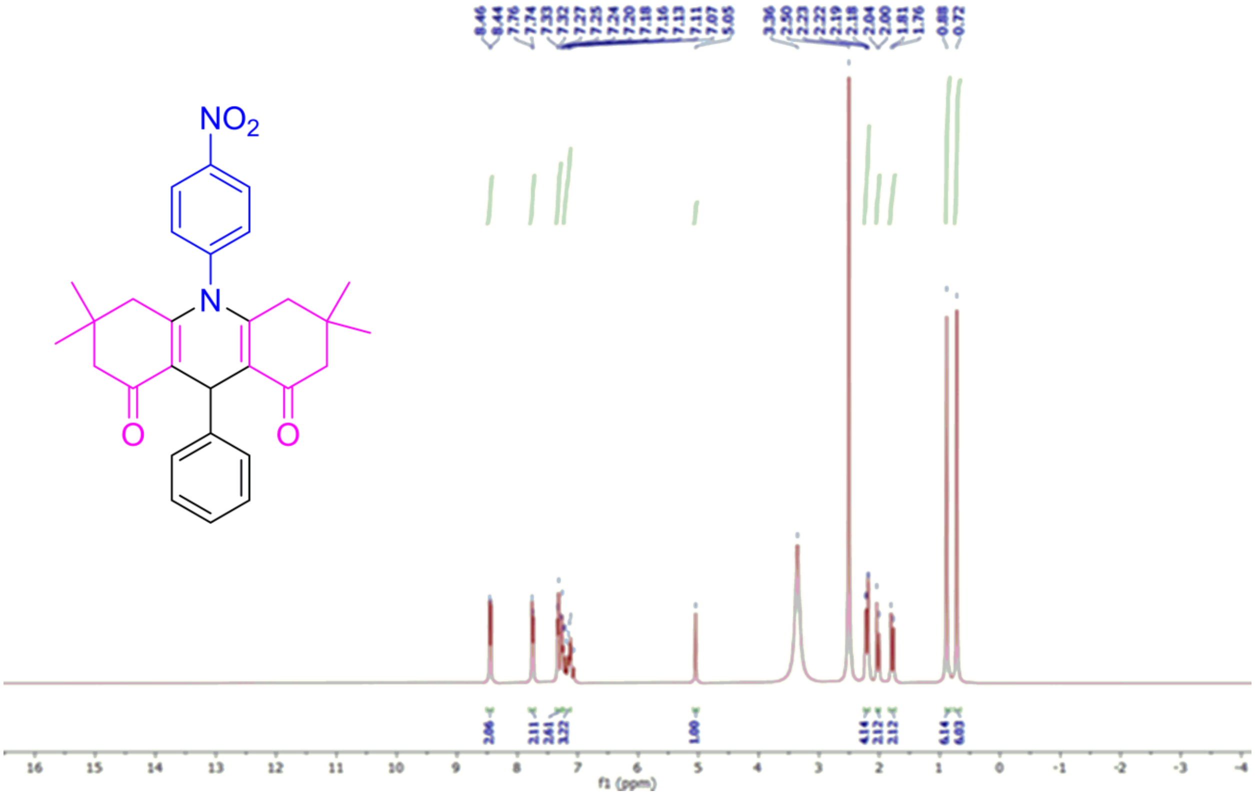

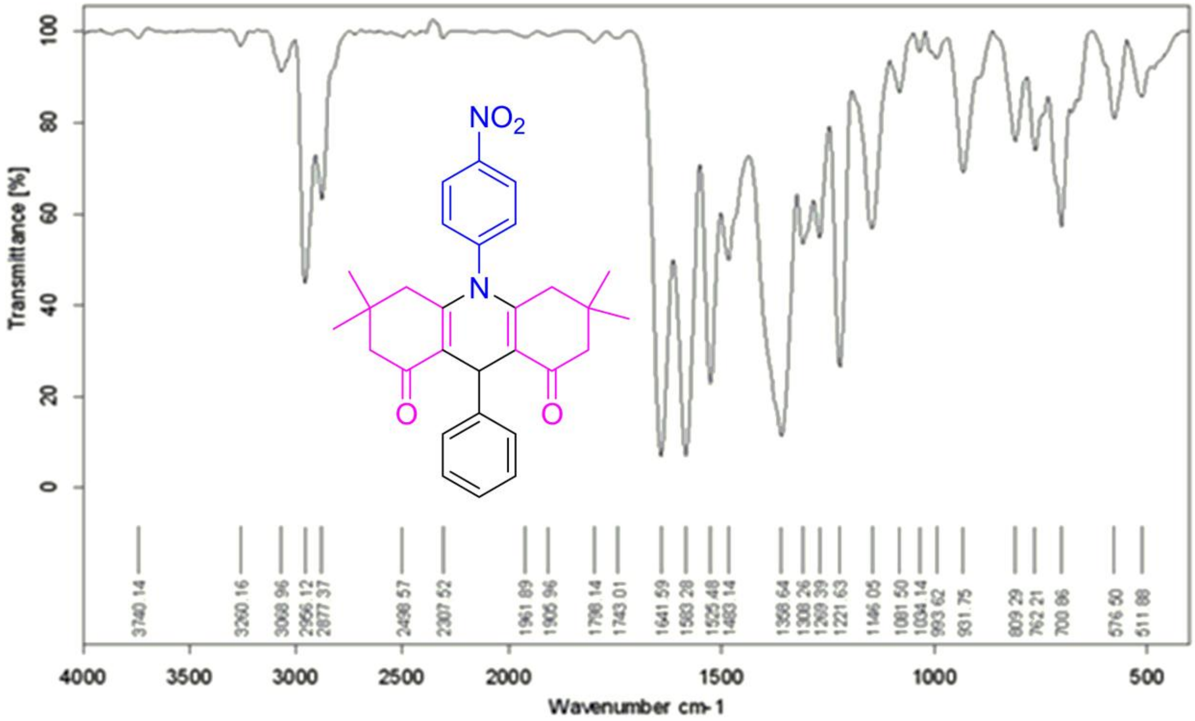

10-(4-bromophenyl)-3,3,6,6-tetramethyl-9-(3-nitrophenyl)-2,3,7,8,9,10-hexahydroacridine-4,5(1H,6H)-dione (4j):

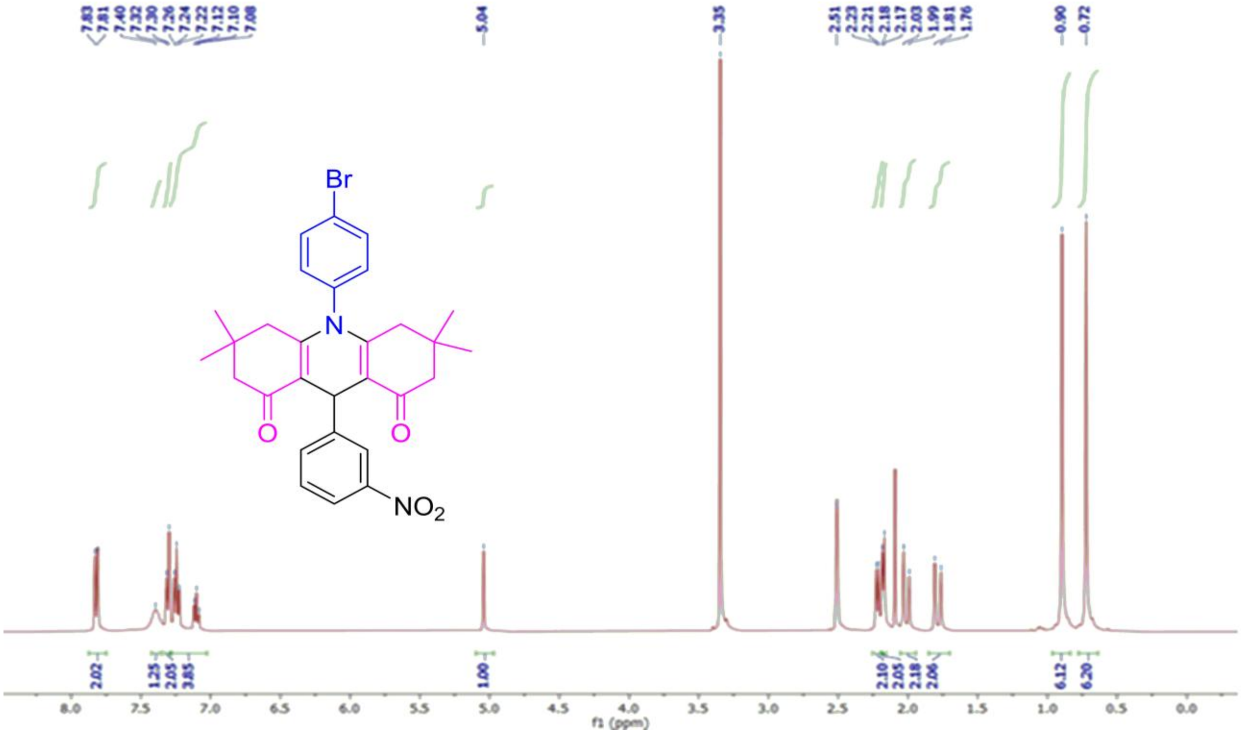

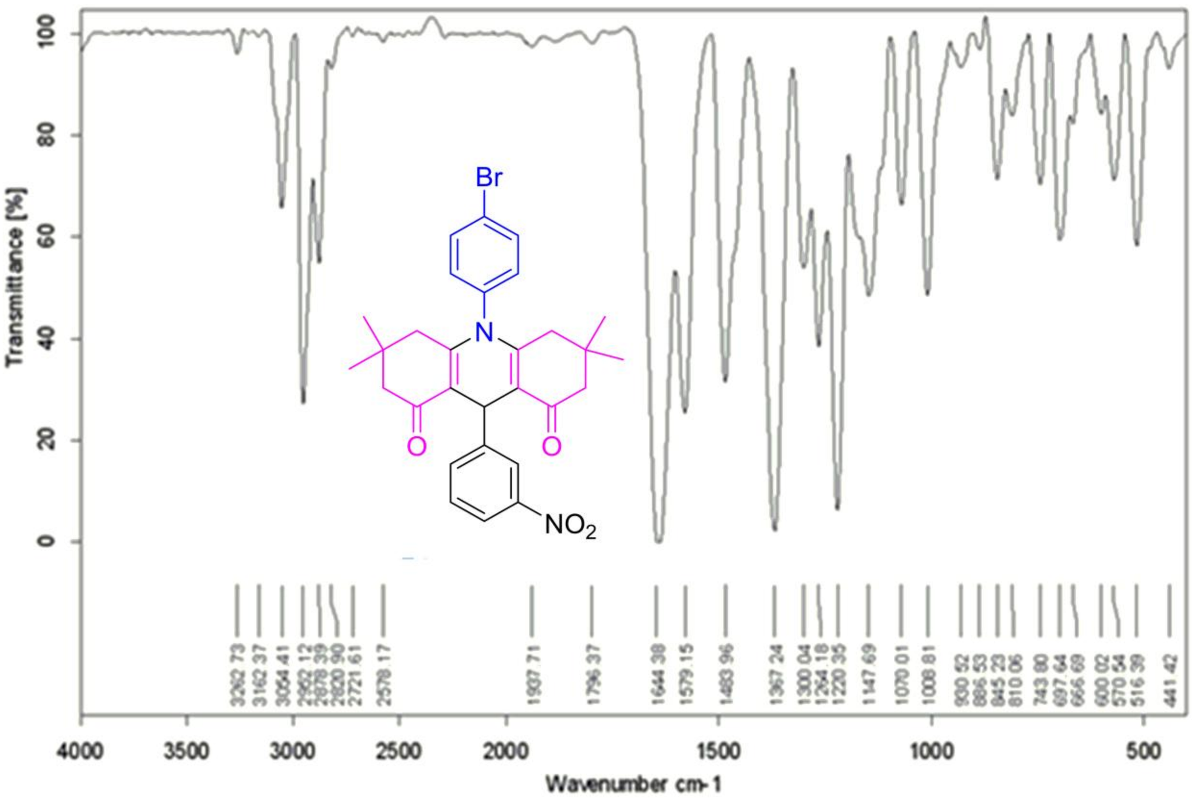

10-(4-bromophenyl)-3,3,6,6-tetramethyl-9-(5-bromo-2-hydroxyphenyl)-2,3,7,8,9,10-hexahydroacridine-4,5(1H,6H)-dione (4k):

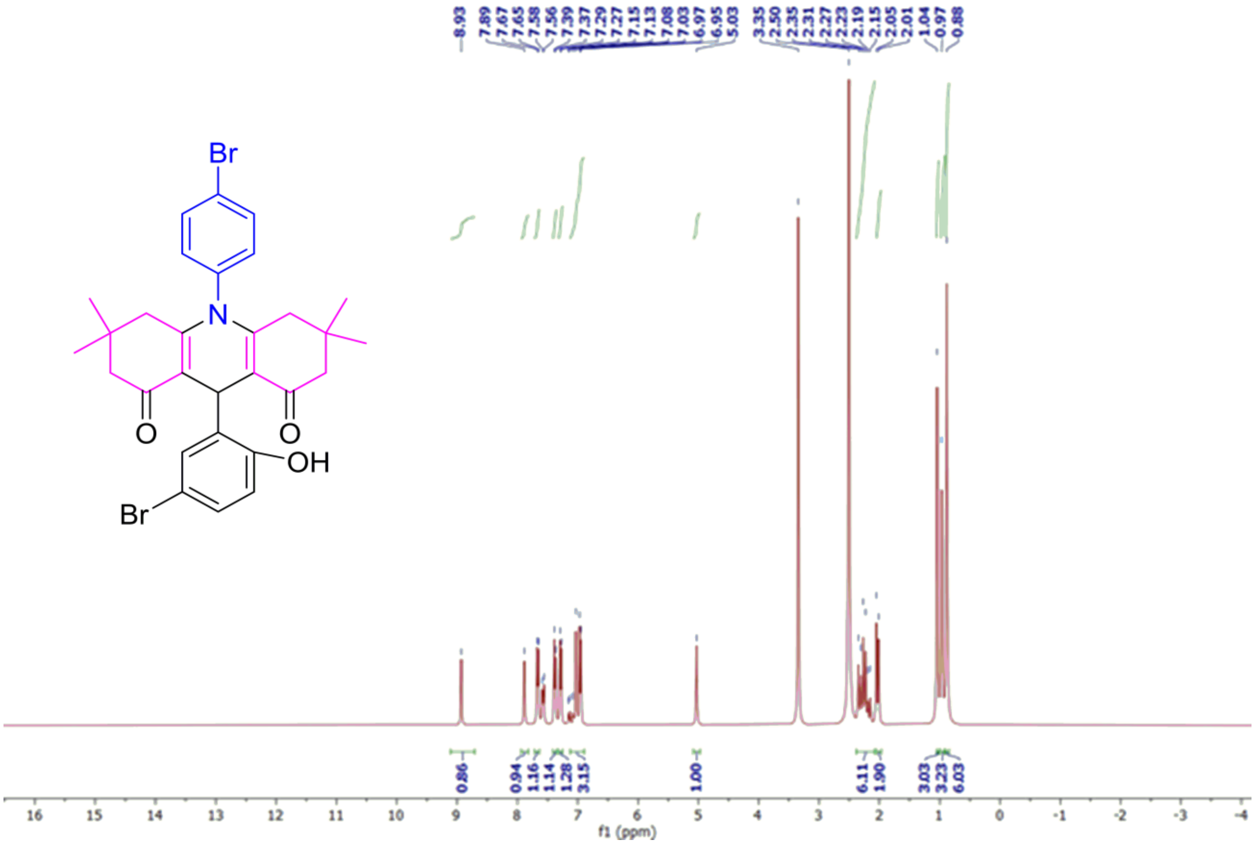

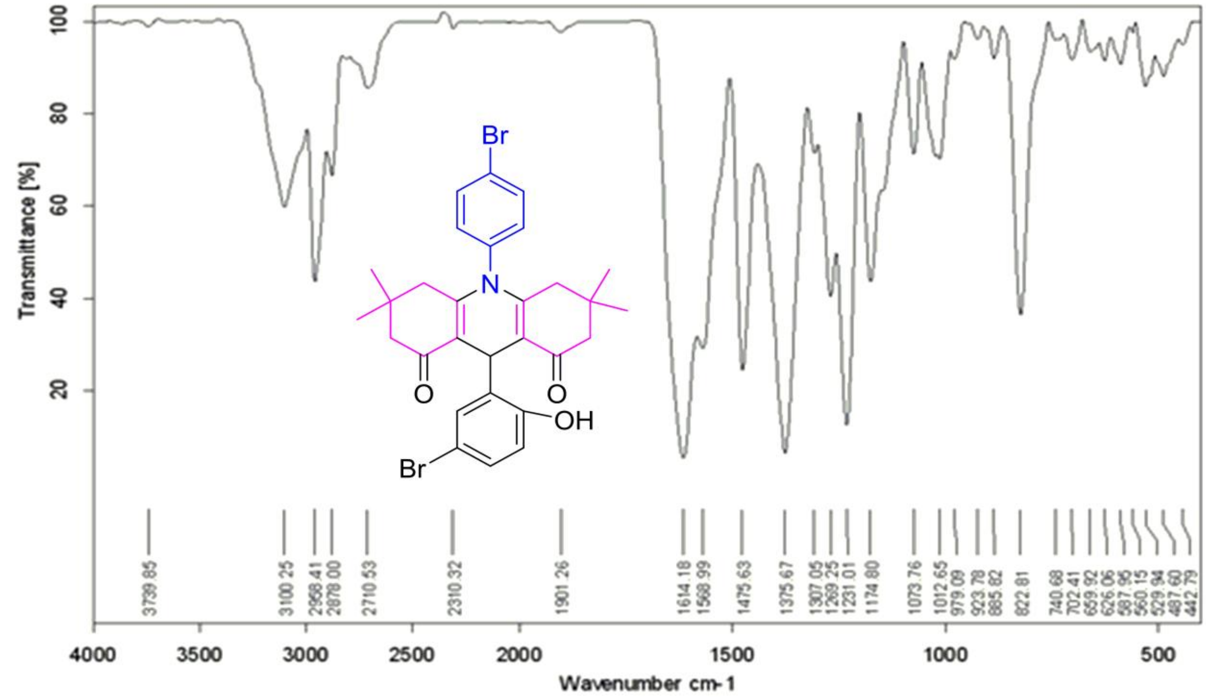

9-(3-nitrophenyl)-3,3,6,6-tetramethyl-10-phenyl-2,3,7,8,9,10-hexahydroacridine-4,5(1H,6H)-dione (4l):

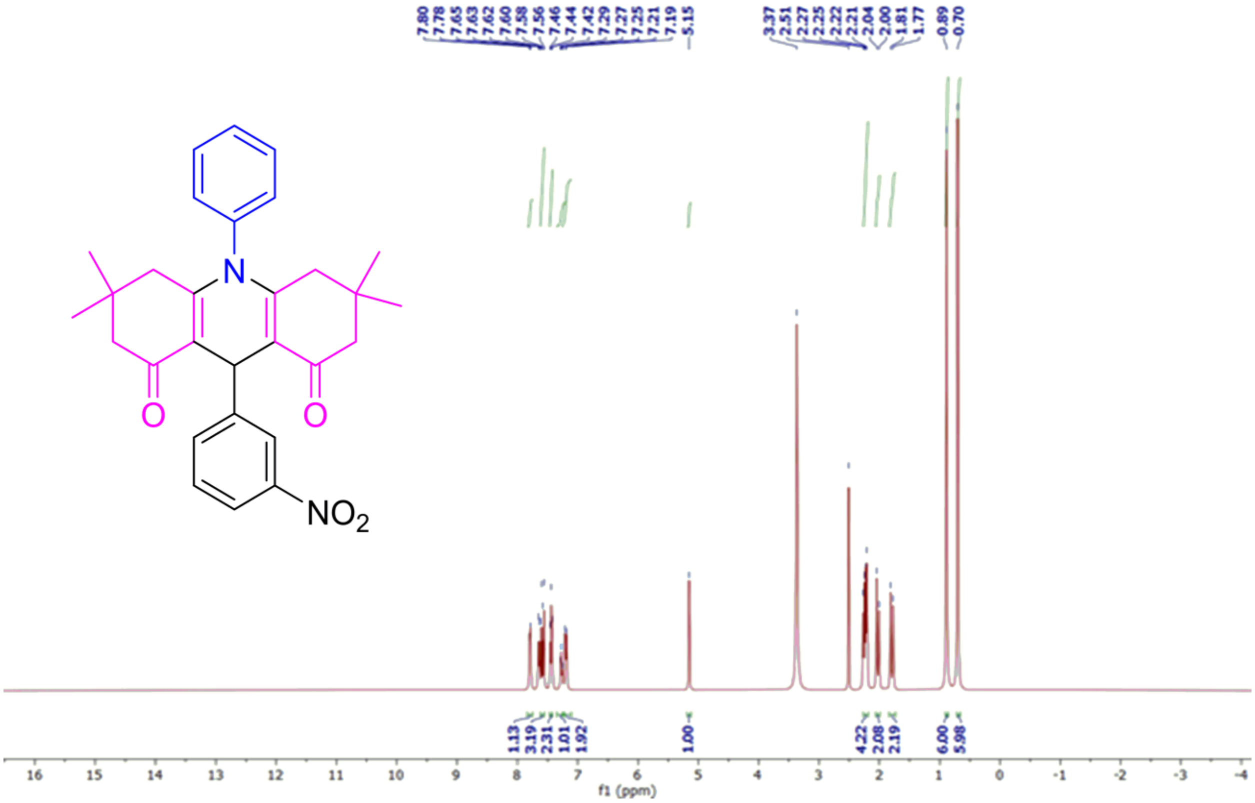

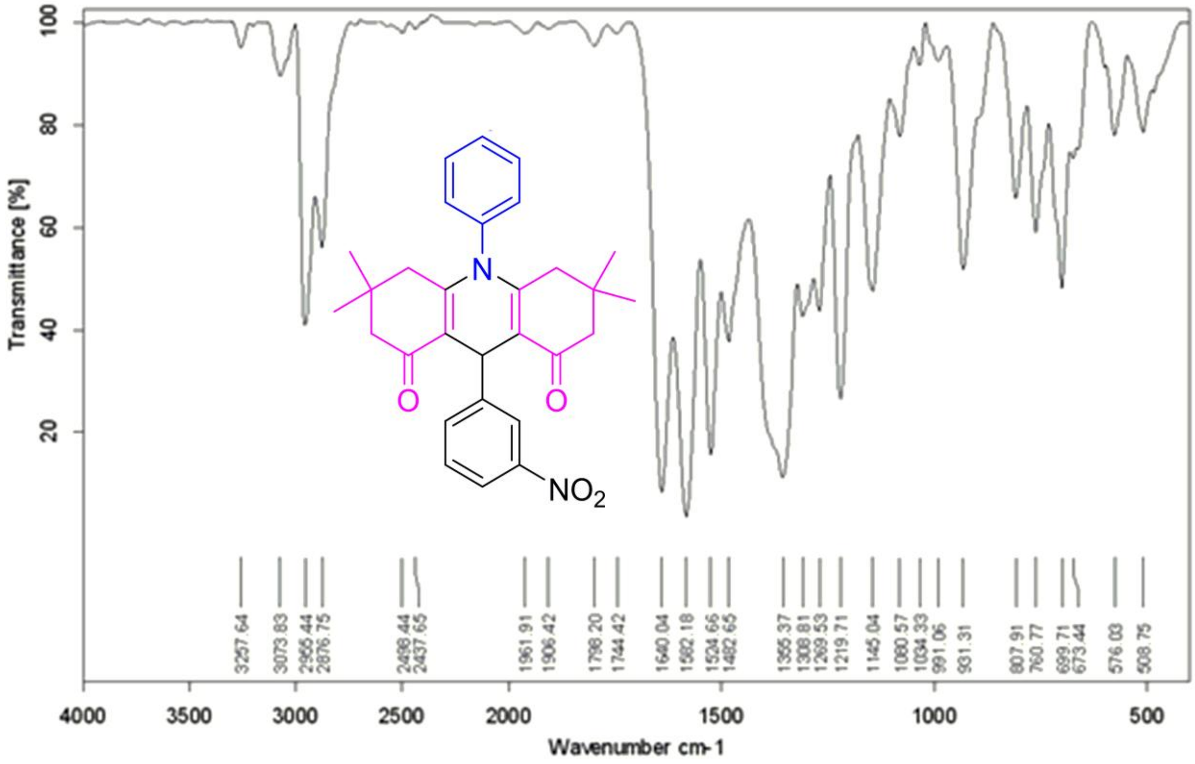

Supplement: Supplementary file 1 — Supplementary Material [file OPEN-15-e202500600-s001.pdf]
